# The “mobile bricks” of human evolution: how transposable elements shaped human phenotypes

**DOI:** 10.3389/fgene.2026.1937765

**Published:** 2026-10-09

**Authors:** Giorgia Modenini, Paolo Abondio

**Affiliations:** 1 Department of Biology, University of Padua, Padua, Italy; 2 Department of Biology, University of Rome Tor Vergata, Rome, Italy

**Keywords:** adaptation, ANCIENT DNA, brain, human evolution, transposable elements

## Abstract

Far from being “junk” and devoid of function, transposable elements have been identified as players in genomes evolution and regulation, and are also used as valuable genetic markers to study population differentiation. Here we present a detailed review on how transposons participated in human evolution: from adaptation to extreme environments and response to both internal and external stressors, to phenotypes directly emerging from transposon mobilization activities. We dedicate a section to brain plasticity and neuronal differentiation, highlighting the possible role of transposable elements in neuro-psychiatric disorders in the context of emerging hypotheses such as the *genomic trade-off* and *price-to-pay*. Furthermore, we discuss methods and approaches to study the influence of transposable elements on human evolution, covering challenges to include ancient DNA in these studies. To conclude, we propose that transposons are not mere “background noise” in host genomes, but that they can be seen as genetic regulators of human phenotypes.

## From genomic junk to functional regulators

Since their discovery in the late 1940s by Barbara McClintock (McClintock, 1950), Transposable Elements (TEs) experienced ups and downs in reputation among scientists. First dismissed as “junk DNA”, “selfish genes” or “parasites” (Orgel and Crick, 1980) because of their mutagenic role, in the last decades they have been recognized as both deleterious (i.e., linked to numerous diseases such as brain disorders and cancers) and beneficial functional objects, for example, in the case of mammalian genome evolution and adaptation (Platt et al., 2018).

In the human genome, TEs are mostly represented by Long Terminal Repeats (LTRs) retrotransposons, non-LTR retrotransposons and, to a lesser extent, sequences derived from DNA transposons. Among non-LTR retrotransposons there are short interspersed nuclear elements (SINEs), long interspersed nuclear elements (LINEs) and the composite family of primate-specific retrotransposons (SVAs).

The vast majority of TEs are now transpositionally incompetent, with only LINE-1s (Cordaux and Batzer, 2009; Levin and Moran, 2011) and possibly HERVs (Human Endogenous Retroviruses, an LTR subfamily) (Autio et al., 2021) that can actively transpose. SINEs - such as *Alu* elements - and the composite family of SVA rely on LINE-1’s machinery to mobilize themselves (Cordaux and Batzer, 2009). However, several studies over the past 20 years suggest that non-active TEs can still play functional roles, for instance as regulatory elements of transcription, gene expression and alternative splicing (Rangwala et al., 2009; Deininger, 2011; Su et al., 2014; Babaian and Mager, 2016; Cao et al., 2020).

In 1990, 40 years after McClintock’s discovery, The Human Genome Project was officially launched, and it was only in 2001, through seminal papers published in both Nature (Lander et al., 2001) and Science (Venter et al., 2001), that the first working draft of the human genome sequence was released, followed by continuous refinements until 2004 (International Human Genome Sequencing Consortium, 2004). Interestingly, since the beginning, repeat sequences - estimated to account for half of the genome - were considered “an extraordinary trove of information about biological processes, [.] holding crucial clues about evolutionary events and forces” (Lander et al., 2001). Lander and colleagues noticed what would have become the standard view on transposable elements and, more in general, repeat sequences, which could “shed light on chromosome structure and dynamics, and provide tools for medical genetic and population genetic studies” (Lander et al., 2001). Furthermore, their initial analysis revealed that ∼45% of DNA sequences in the human genome are ascribable to TEs, a figure further increased by the most recent T2T human genome assembly (Nurk et al., 2022), in which 54% of genomic DNA is estimated to be derived from TEs and repetitive elements (Hoyt et al., 2022).

In this work, we will review the current literature about TEs regulatory functions in humans, and reinforce the view that transposons, despite being potentially harmful for the host genome, can also positively impact its evolution. Rather than considering transposable elements only as sources of genomic instability or as passive genetic components, we discuss them here as potential mediators connecting genomic variation to phenotypic innovation. From this perspective, their evolutionary relevance can be considered at multiple levels: the generation of genetic and structural variation, the recruitment of TE-derived sequences into gene-regulatory networks and the emergence of phenotypic consequences. We use examples spanning population adaptation, phenotypic innovation and brain development to illustrate these different levels, while also highlighting where the current evidence remains correlative or technically limited. In particular, the potential contribution of TEs to human brain evolution represents an important unresolved question, because establishing a causal link between TE-derived variation, changes in neural development or function and cognitive phenotypes remains challenging.

## Transposable elements activities in the human genome

In the last decades, the impact of TEs on the host genome has been the focus of intense research, and it has been shown that TE insertions can generate diversity in various ways, being both positive and detrimental players in human genome’s evolution [reviewed in (Reilly et al., 2013; Bourque et al., 2018; Gebrie, 2023)].

Growing evidence points toward an important role of TEs in shaping evolutionary and adaptive processes, such as (but not limited to): generation and transcriptional regulation of genes and pseudogenes (Moran et al., 1999; Chuong et al., 2017), somatic mosaicism (Muotri et al., 2005; Baillie et al., 2011; Evrony et al., 2012), increase in complexity and evolution of gene regulatory networks (Feschotte, 2008) and alteration of epigenetic mechanisms (Fedoroff, 2012).

Most of TEs transposition activity is repressed by the host genome via several mechanisms, including DNA and RNA methylation, histone modifications, Piwi-interacting RNAs (piRNAs) and small interfering RNAs (siRNAs) (Copley and Shorter, 2023). Different Authors suggest that histone modifications may play a more important role in suppressing evolutionarily older TEs, whereas DNA methylation seems to act preferentially on evolutionarily young TEs (Jönsson et al., 2019; Ewing et al., 2020), such as *Alu*, LINE-1 and SVA. Therefore, to survive this strong restriction activity, new TE copies must appear in the germline, as somatic transposition events are, by definition, peculiar of the individual itself and are not inherited by further generations. Interestingly, despite being a strong mutagenic source and therefore being strictly under control during embryonic development, TEs have been shown to be de-repressed during specific developmental stages (Friedli and Trono, 2015; Gerdes et al., 2016; Percharde et al., 2018; Senft and Macfarlan, 2021; DiRusso and Clark, 2023): for example, HERV-K transcription in human embryogenesis starts during embryonic genome activation at the eight-cell stage and remains until the blastocyst stage (Gerdes et al., 2016).

As the Author stated in his article (Feschotte, 2008), “in spite of and, to some extent, because of this selfish and parasitic nature, the movement and accumulation of transposable elements have exerted a strong influence on the evolutionary trajectory of their hosts”. TE-derived sequences have been, in some cases, co-opted to serve host cellular functions in a process called *exaptation* (Joly-Lopez and Bureau, 2018; Modzelewski et al., 2022; Gasparotto et al., 2023; Oomen and Torres-Padilla, 2024). TE sequences can, for instance, serve as *cis* regulatory elements of gene expression and alternative splicing (Chuong et al., 2017; Cao et al., 2020). This function arises from the very nature of TEs: mobilization-competent elements rely on the host’s molecular machinery to transpose, therefore they have evolved *cis*-regulatory sequences that mimic host promoters (Chuong et al., 2017). In fact, it has been recently discovered that TEs can create alternative transcription start sites (TSSs; Table 1) (Gu et al., 2024) and that 18%–31% of human TSSs overlap with TEs (Faulkner et al., 2009; Djebali et al., 2012). TEs also provide a major promoter source for long noncoding RNAs (lncRNA; Table 1) [reviewed in (Fort et al., 2021)]: lncRNAs derived from TEs play a crucial role in diseases, mostly in cancer [reviewed in (Reggiardo et al., 2022; Zhang, 2024)], by modulating gene expression, influencing chromatin structure, and interacting with RNA-binding proteins (Nguyen et al., 2020; Prokopov et al., 2025). More than 80% of lncRNAs contain TE sequences, with these making up to 30%–42% of lncRNA sequence lengths (Kelley and Rinn, 2012; Kapusta et al., 2013).

**TABLE 1:** Synthesis of the most studied TE-related processes and influences on the human genome.

TE-related process	Mechanism	TEs/genomic context	Functional consequence	Species
Epigenetic repression	DNA methylation	Predominantly young TEs, including Alu, LINE-1 and SVA	Suppression of TE transcription and mobilization	Human/mammals
Epigenetic repression	Histone modifications	Predominantly evolutionarily older TEs	Chromatin-mediated TE silencing	Human/mammals
Post-transcriptional repression	piRNAs/siRNAs	Transposable-element transcripts	RNA-mediated suppression of TE activity	Animals
Developmental de-repression	Transient TE transcription	HERV-K and other TEs	TE activity during specific embryonic stages	Human
Exaptation	Co-option of TE sequences	Multiple TE families	Recruitment of TE-derived sequences for host functions	Multiple species
Transcriptional regulation	Promoters/enhancers	Alu, LINE-1, SVA and other TEs	Regulation of neighboring genes	Human/mammals
Transcription initiation	Alternative TSSs	TE-derived promoter sequences	Generation of alternative transcripts	Human
RNA processing	Alternative splicing	TE-derived regulatory sequences	Transcript isoform diversification	Human/mammals
lncRNA evolution	TE-derived promoters/sequences	Multiple TE families	Formation and regulation of lncRNAs	Human/mammals
Disease-related regulation	TE-derived lncRNAs	Multiple TE families	Altered gene expression and chromatin regulation, particularly in cancer	Human

Taken together, these observations establish that TE-derived sequences can acquire functional roles in the human genome, particularly through their contribution to transcriptional and epigenetic regulation (Table 1). However, the presence of a TE-derived regulatory element does not necessarily demonstrate that it has been evolutionarily selected or that it produces a measurable phenotypic effect. An important unresolved question is, therefore, how frequently TE-derived regulatory functions translate into adaptive phenotypes, and which genomic, epigenetic and evolutionary features distinguish functional co-option from neutral or deleterious TE persistence.

## Emerging methods and approaches to study transposable elements impact on human evolution

As more and more data of both whole genomes and TE polymorphisms/sequences are available - see for instance the well-established use of the Repbase (Bao et al., 2015) and Dfam databases (Storer et al., 2021) (Figure 1A; Table 2), now unified (Kojima et al., 2026) - novel approaches to study TEs impact on human evolution are emerging: for example, the Zoonomia project is an international collaboration to discover the genomic basis of shared and specialized traits in mammals, including humans (https://zoonomiaproject.org/). By comparing 241 mammalian genomes, Andrews and colleagues (Andrews et al., 2023) discovered that about 20% of the analyzed transcription factor binding sites (TFBSs) are derived by transposable elements and exhibit intricate patterns of gains and losses during primate evolution (Andrews et al., 2023). The Zoonomia comparative approach is able to discriminate between *conserved* and *innovative* sequences in mammalian genomes, eventually highlighting the role of TEs in human evolution compared to other mammals (Figure 1B).

**FIGURE 1:**
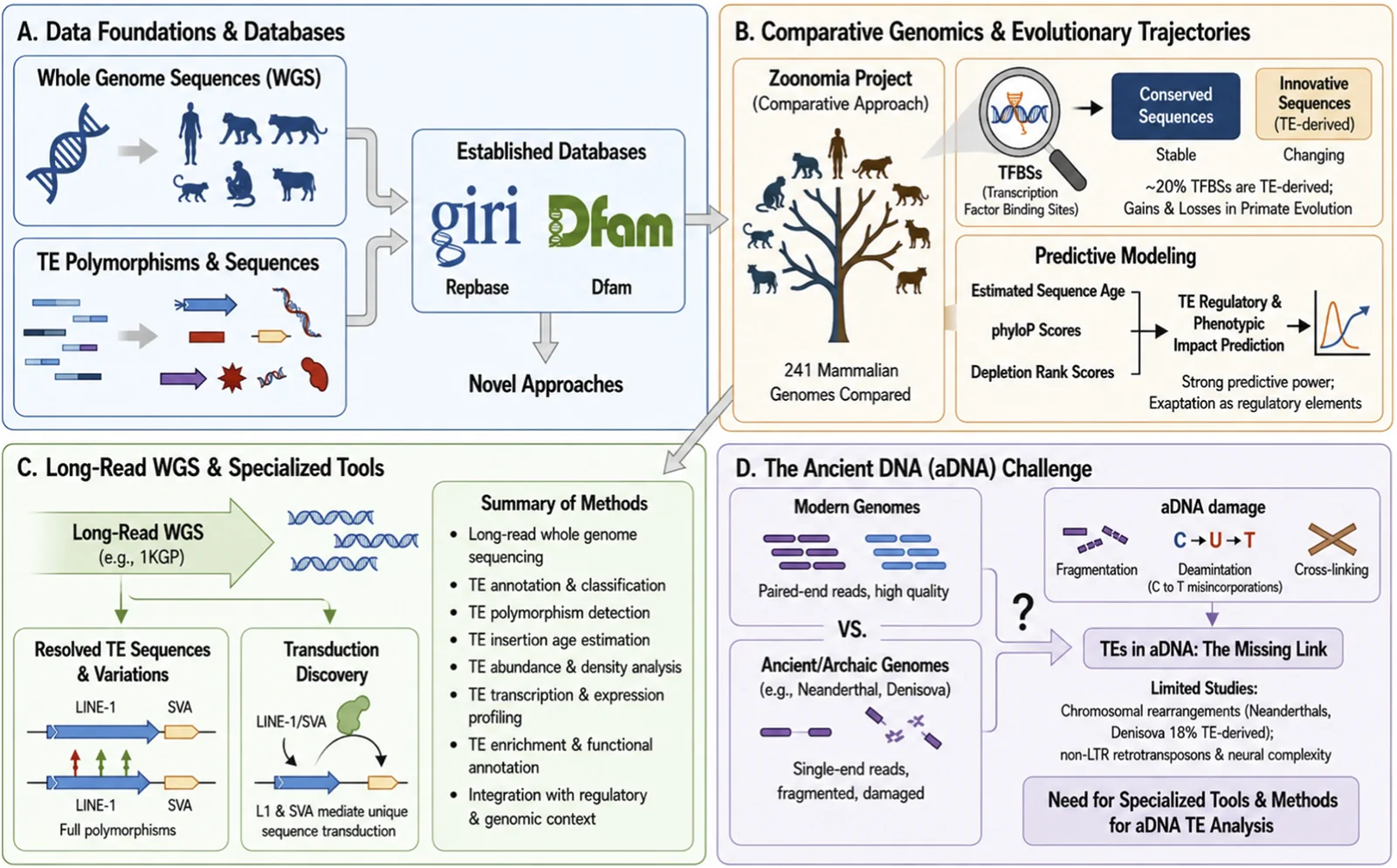
Emerging methods and approaches to study TEs impact on human evolution. Unveiling transposons impact on the human genome is still challenging. However, recent advances in sequencing technologies and tools to analyze TEs **(A, C)** are helping scientists to elucidate TEs regulatory activity on genes and genomes and to better characterize TE insertions/sequences **(B)**. Nevertheless, improvements must be made, for instance in designing tools capable of leveraging aDNA and therefore investigating the TE content of ancient genomes **(D)**. Created with biosketch.com.

**TABLE 2:** Well-established databases for studying and annotating transposable elements.

Databases	Content	Typical application
Dfam (https://www.dfam.org/home)	Hidden Markov models (HMMs) for TE families	Standard reference database for RepeatMasker
RepBase (https://www.girinst.org/repbase/update/browse.php)	Curated TE consensus sequences	Historical reference database (restricted license)
UCSC repeat annotation (https://genome.ucsc.edu/cgi-bin/hgTables)	Repeat annotations for the human genome	Comparative genomic analyses
gnomAD-SV (https://gnomad.broadinstitute.org/)	Structural variants and mobile element insertions	Population allele frequency estimation
1000 genomes MEI callset (https://doi.org/10.1101/gr.218032.116)	Polymorphic mobile element insertions	Population genetics and evolutionary studies

In a recent unpublished work, Cerruti and colleagues (Cerruti et al., 2024) used a similar comparative approach to predict the regulatory and phenotypic impact of human TEs: by taking advantage of estimated sequences age (Marnetto et al., 2018) and using both phyloP (Zoonomia Consortium, 2020) and depletion rank scores (Halldorsson et al., 2022), the Authors suggest that “TE sequence and conservation features are strongly predictive of their functional impact on the host phenotype” and that these effects “are largely driven by their exaptation as regulatory elements and the ensuing changes in gene expression” (Cerruti et al., 2024) (Figure 1B).

Exceptional improvements have been made in the last decade thanks to the advent of long-read whole genome sequences (WGS), which not only enable a better identification of TEs, but also allow to disentangle variations and functionality of their resolved sequences. For instance, Schloissnig and colleagues generated more than 1,000 long-read WGS of individuals from the 1KGP (Figure 1C), allowing not only for reconstruction of polymorphic TE sequences, but discovering that LINE-1 and SVA retrotransposition activities frequently mediate transductions of unique sequences (Schloissnig et al., 2025).

These improvements have been possible since in the last decades several tools for detecting, characterizing and annotating TEs in human genomes have been published. Some of the most widely used are the Mobile Element Locator Tool [MELT (Gardner et al., 2017):], McClintock (Nelson et al., 2017; Chen et al., 2023) and xTea (Chu et al., 2021), among several others (Table 3). MELT is specifically designed to cope with human high-coverage Illumina short-reads WGS and it is based on TE reference sequences; it allows detection and genotyping of TE polymorphisms and it was tested on 1000 Genomes Project sequences (Gardner et al., 2017). xTea is a Python-based tool able to detect TEs in both short- and long-read WGS; TEs are then genotyped with a machine-learning-based method (Chu et al., 2021). Instead, McClintock is a meta-pipeline, integrating several previously published tools with specific dependencies, run interfaces and output formats for TE detection and annotation, allowing users to reproducibly install, execute and evaluate multiple TE detectors with outputs generated in standardized formats (Nelson et al., 2017; Chen et al., 2023) (Table 3).

**TABLE 3:** Tools for detecting TEs in both short- and long-read WGS.

Discovery and detection tools	Input → output	Supported technology	Links
RetroSeq	BAM → VCF	Illumina	https://github.com/tk2/RetroSeq
TEBreak	BAM → candidate insertion list	Illumina	https://github.com/adamewing/tebreak
Tangram	BAM → VCF	Illumina	https://github.com/jiantao/Tangram
TEFLoN	BAM → Allele frequencies and genotypes	Illumina	https://github.com/jradrion/TEFLoN
McClintock2	FASTA/FASTQ → Combined outputs	Illumina	https://github.com/bergmanlab/mcclintock
STEAK	BAM → HERV insertion calls	Illumina	https://github.com/applevir/STEAK
MELT	BAM/CRAM → VCF	Illumina short reads	https://melt.igs.umaryland.edu/
xTea	BAM → VCF	Illumina, PacBio, Oxford Nanopore	https://github.com/parklab/xTea
Sniffles2	BAM → VCF	Long reads	https://github.com/fritzsedlazeck/sniffles
nanotei	FASTQ/BAM → TE insertion calls	Oxford Nanopore	https://github.com/Kirovez/nanotei
TLDR	BAM → Annotated insertions	Oxford Nanopore, PacBio	https://github.com/adamewing/TLDR
PALMER	PacBio BAM → Complete insertion sequences	PacBio	https://github.com/WeichenZhou/PALMER

The TEToolkit Suite (https://www.mghlab.org/software), which includes four different tools, namely, TEtranscripts (Jin et al., 2015), TElocal (a locus-specific version of TEtranscripts), TEsmall (O’Neill et al., 2018), and TEsingle (Forcier et al., 2026), is a very useful suite to analyze TEs in RNA-seq datasets (Table 4).

**TABLE 4:** Tools for the analysis of RNA-seq data and quantification of TEs expression.

Expression tools	Application	Input	Supported technology
TEtranscripts (https://www.mghlab.org/software/tetranscripts)	Differential TE expression analysis	BAM + GTF	Bulk RNA-seq
SQuIRE (https://github.com/wyang17/SQuIRE)	End-to-end TE expression pipeline	FASTQ/BAM	Bulk RNA-seq
TElocal (https://www.mghlab.org/software/telocal)	Locus-specific TE quantification	BAM	Bulk RNA-seq
SalmonTE (https://github.com/jeonglab-bcm/SalmonTE)	Fast TE expression quantification	FASTQ	Bulk RNA-seq
L1EM (https://github.com/FenyoLab/L1EM)	LINE-1 expression quantification	BAM	RNA-seq
scTE (https://github.com/JiekaiLab/scTE)	Single-cell TE expression analysis	FASTQ	scRNA-seq
IRescue (https://github.com/bodegalab/irescue)	TE quantification in single-cell RNA-seq	BAM	scRNA-seq

Furthermore, the regulatory and phenotypic impact of TEs on the human genome is the focus of several tools and softwares published in the last few years (Figure 1C). For instance, an R package called TEffectR enables to study the potential influence of TEs on proximal gene expression from a given RNA-sequencing dataset (Karakülah et al., 2019), allowing scientists to understand potential impacts of TE expression on the regulation of nearby genes. TE Density (Teresi et al., 2022) is a tool able to investigate TE biology and density inside or near genes. Results and data can be used to examine TE presence genome-wide, between genomes, and at the individual gene scale. Data “can be used as a screen to examine changes in TE presence, or as part of a larger analysis incorporating other datasets such as methylation or gene expression data” (Teresi et al., 2022) (Figure 1C). TEENA (Transposable Element ENrichment Analysis: https://sun-lab.yzu.edu.cn/TEENA/) is a web/local tool to perform analysis of TEs enrichment in both model and non-model organisms (Li et al., 2024). Taking genomic interval data as the only input, it allows to identify the enriched TEs in distinct genomic intervals - for example, a set of enhancers or TFBSs - as candidates for further studies (Li et al., 2024) (Figure 1C). Of course, more tools and softwares with the same purposes have been published, and these are just a few examples, as a detailed description of methods and tools is not the focus of this review.

Far from being an exhaustive list, all of the tools mentioned here allow to study TEs regulatory functions. However, one important question arises: how can scientists unveil TEs impact on human evolution if they do not take into consideration ancient DNA (aDNA) and analyze ancient/archaic genomes to estimate TEs evolutionary trajectories? In a recent and exceptional work, Wang and colleagues (Wang et al., 2021) thoroughly compare the possible strategies to study TEs in aDNA and, more specifically, their impact on human brain evolution. They highlight that only a few studies have been published, due to the lack of specialized tools to investigate the TE content of aDNA (Figure 1D). Nevertheless, the number of ancient and archaic human genomes available for analysis has continued to increase, certainly providing a significant opportunity to reconstruct the dynamics of TE insertions and their contribution to human evolution. So far, to our knowledge, the most notable works in this field are those from (Rogers, 2015; Gardner et al., 2017; Guichard et al., 2018). In Rogers’ work, the Author identifies chromosomal rearrangements in archaic hominins that were partially due to transposition activity: 13% of these genomic structural changes in Neanderthal are transposition-derived, whereas in Denisova 18% of these changes are ascribable to TEs activity (Rogers, 2015). Interestingly, Guichard and colleagues, who focused on the comparative analysis of non-LTR retrotransposons between modern humans, archaic individuals and chimpanzees, highlighted that these TEs “impacted our differentiation as a species, most likely inducing an increase in neural complexity, and have been a constant source of genomic variability all throughout the evolution of the human lineage” (Guichard et al., 2018). However, especially due to technical issues when dealing with aDNA, the study of TEs in ancient *Homo* is still in its infancy, but at the same time necessary (Wang et al., 2021). Indeed, the authors of these studies all used aDNA from archaic individuals (Neanderthal and Denisova), but not from ancient *Homo sapiens*. This is possibly due to several technological and methodological limitations: 1) every method for the analysis and identification of human polymorphic TEs is designed to cope with modern genomes, that are usually paired-end sequences - while ancient DNA usually comes with high proportions of single-end sequences; 2) due to their repetitive nature, transposable elements are often cut out from the sequence during the alignment to a reference genome; 3) ancient DNA is characterized by low percentage of endogenous ancient human DNA, high degradation and frequent sequence alterations due to cytosine deamination (Wang et al., 2021). As reported by Briggs (Briggs et al., 2007), cytosines in ancient DNA can undergo deamination and be converted into uracils, which are subsequently sequenced as thymine. This process is known as “C-to-T misincorporation” and, in double-stranded DNA libraries, is also observed as G-to-A misincorporation. Because aDNA is exposed to external agents and undergoes various taphonomic processes, it accumulates a high number of base-pair substitutions that can alter the original DNA sequence. More broadly, aDNA damage includes “the fragmentation of DNA molecules into ultra-short DNA fragments, the conversion of the four nucleotides into various derivatives and the cross-linking of DNA to other molecules” (Orlando et al., 2021).

These post-mortem modifications are particularly relevant for TE analysis because they can generate mismatches close to insertion breakpoints, interfere with read mapping and potentially mimic sequence variation. Early damage-aware approaches such as *snpAD* and *AntCaller* have demonstrated that explicitly modelling aDNA damage can substantially improve the reliability of variant inference from ancient genomes (Zhou et al., 2017; Prüfer, 2018). A future direction is therefore the development of aDNA-aware TE callers capable of jointly modelling TE-specific mapping patterns, short fragment lengths, single-end data and post-mortem damage. Current TE callers (Table 3) have been primarily designed and benchmarked for modern human sequencing data, but their underlying strategies, particularly the detection of split and discordant reads and the characterization of insertion breakpoints, provide a framework that can be adapted to ancient genomes. Such future callers could explicitly incorporate position-dependent C-to-T/G-to-A damage probabilities and fragment-length distributions into candidate scoring, rather than treating damaged bases as conventional sequencing errors. Simulations using aDNA-specific damage profiles, for example, with tools such as *gargammel* (Renaud et al., 2017), could further enable systematic benchmarking of TE detection sensitivity and false-positive rates across different levels of endogenous DNA, fragment length, coverage and damage. More recently, TE-specific simulation frameworks such as *TEvarSim* (Miao and Li, 2026) have been developed to generate realistic datasets containing TE insertions and deletions, potentially facilitating the development and benchmarking of such aDNA-oriented approaches.

A second promising direction concerns read alignment. Conventional mapping strategies can introduce substantial reference bias, particularly for short and damaged aDNA reads (Martiniano et al., 2020; Vaddadi et al., 2026). Recent benchmarking has shown that the choice of aligner and mapping-quality threshold can strongly influence the representation of divergent sequences in ancient genomes, with particularly pronounced effects for reads shorter than approximately 80 bp (Oliva et al., 2021). Consequently, improved single-end alignment strategies, supported by pangenome graphs, that explicitly account for deamination and very short fragments could increase the number of informative reads available for TE detection. For example, SAFARI (Rubin et al., 2025) introduces a damage-aware pangenome alignment framework that uses purine/pyrimidine-encoded seeds to rescue alignments containing deaminated bases, improving alignment accuracy for damaged ancient sequences. Although SAFARI was not developed specifically for TE detection, its conceptual framework could be valuable for repetitive regions, where loss of a small number of informative reads may prevent the identification of an insertion. Similarly, pangenome-based approaches to TE genotyping have demonstrated that representing TE variation beyond a single linear reference can improve the detection and genotyping of TE insertions and deletions in short-read data (Groza et al., 2023; 2024; Sierra and Durbin, 2024).

A combination of these strategies could be particularly useful for distinguishing genuine ancient TE insertions from apparent variants generated by deamination or misalignment to other repetitive elements. The possibility of reconstructing ancestral genomic states may further improve TE annotation, as recent work has shown that ancestral genome reconstruction can help identify highly diverged and otherwise difficult-to-annotate TE-derived sequences (Matsushima et al., 2024). These developments are especially relevant given that the first applications of TE profiling to archaic human genomes have already demonstrated its feasibility. For example, recent analyses have successfully applied existing TE-insertion detection strategies to the study of archaic humans together with ancient and modern individuals from the Tibetan Plateau, illustrating that the methodological gap is beginning to narrow (Modenini et al., 2024). Moreover, the increasing availability of high-coverage ancient human genomes (Mallick et al., 2024) provides a growing temporal series against which TE insertion frequencies and evolutionary trajectories can be reconstructed.

## Transposable elements and phenotypic adaptability

### Adaptations to extreme environmental conditions

One of the most interesting features of *Homo sapiens* evolution is being capable of adapting to new and extreme environments in every continent (except for Antarctica). For instance, several populations have convergently adapted to the hypoxic conditions of high-altitude in the Himalayas of South-Central Asia (Tibetans, Sherpas and Ladakhis), in the Andes of South America (Andeans), and in the highlands of Eastern Africa (Ethiopians) (Witt and Huerta-Sánchez, 2019). A well-known case concerns adaptive mutations and introgressed variants in *EPAS1* (endothelial PAS domain protein 1), *EGLN1* (egl-9 family HIF 1) and *PPARA* (peroxisome proliferator-activated receptor alpha), all of which are part of the hypoxia-inducible factor (HIF) pathway (Figure 2), in Tibetan populations (Beall et al., 2010; Simonson et al., 2010; Yi et al., 2010).

**FIGURE 2:**
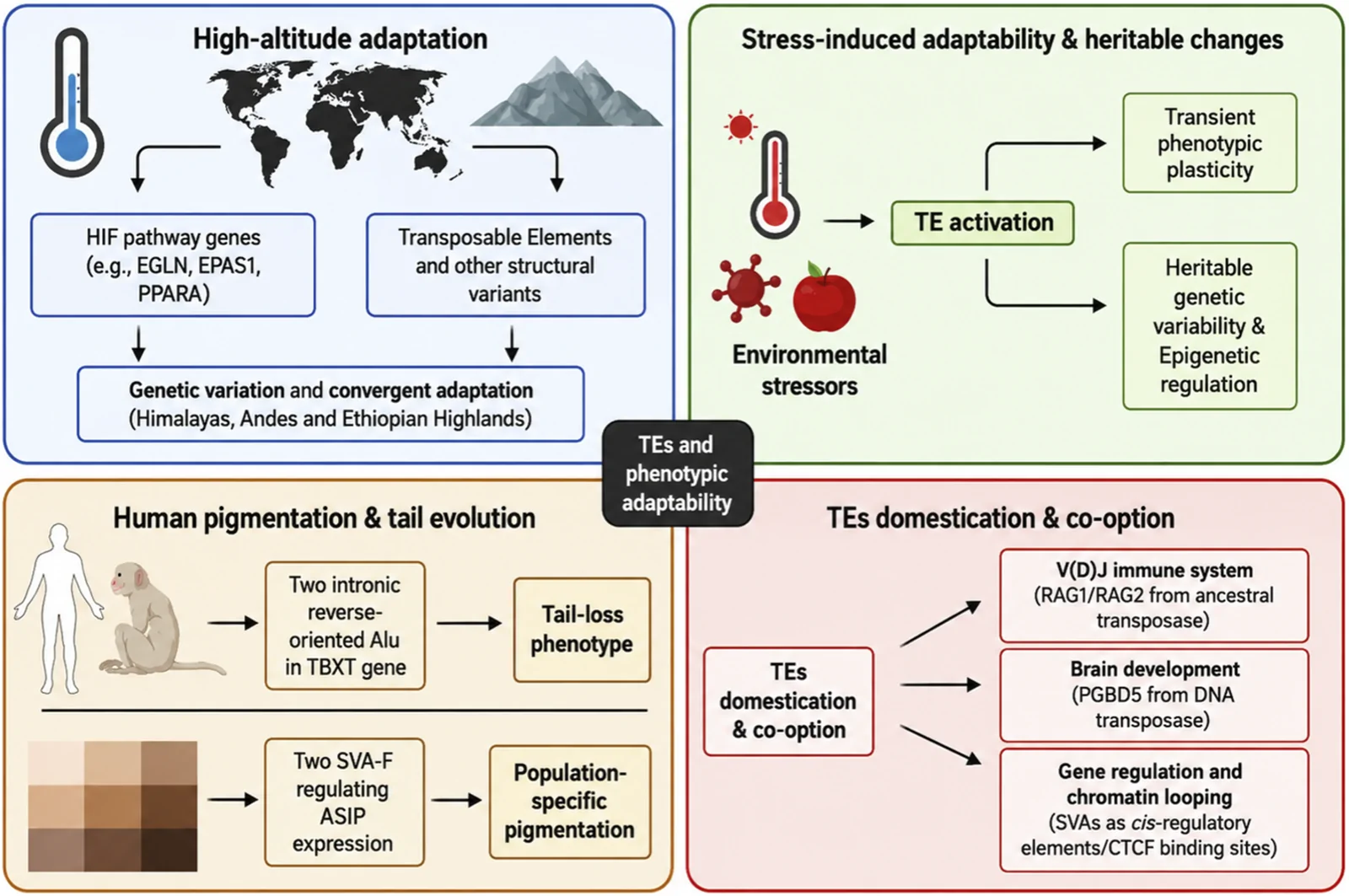
Structural variants such as transposable elements have been implicated in several phenotypic changes during human life (such as stress response) and evolution, from adaptation to high altitude, to skin/hair pigmentation and tail-loss evolution. They have also been domesticated to serve host cellular functions and became important contributors for immune system’s evolution. Created with biorender.com.

Few studies exist on the role of structural variants (SVs) in the process of adaptation to high altitude: for example, Shi and colleagues, using long-read sequencing of 320 Han Chinese and Tibetan genomes, identified more than 136 thousand SVs, expanding the current knowledge about genetic variation in Asian populations (Shi et al., 2023).

Another work, focused on polymorphic mobile elements, investigated the possible influence of evolutionarily recent TEs on Sherpa and Tibetan adaptation to high altitude (Modenini et al., 2024). In this work, we identified more than 12 thousand polymorphic TEs in 118 modern, ancient and archaic individuals, highlighting the putative contribution of the non-coding genome in high-altitude adaptation and giving also a glimpse inside SVs evolutionary trajectories in high-altitude populations (Modenini et al., 2024). Moreover, a high number of TEs seem to discriminate, in terms of presence/absence and allele frequencies, between high- and low-landers, revealing a possible role in the control of peculiar genes involved in oxidative stress and hypoxia-induced inflammation, which themselves modulate the expression or translation of other genes (Modenini et al., 2024).

#### From stress-induced adaptability to heritable genetic changes

Transposable Elements serve as a substrate for stress-induced genomic variability, through what may represent an adaptive toolkit for organisms - including *Homo* - to respond to their environments at both individual and evolutionary scales (Hunter, 2020). A growing body of research is indeed highlighting how environmental stressors can trigger TE activity, facilitating rapid genomic shifts that may entail adaptive responses (Piacentini et al., 2014) (Figure 2). In particular, TEs can be seen as molecular intermediaries that embed transient phenotypic plasticity into heritable genotypic variability and are activated under conditions of stress, such as changes in temperature, nutrition or exposure to toxins (Pimpinelli and Piacentini, 2020). For example, laboratory experiments with human neuroblastoma cell lines exposed to variable concentrations of heavy metals have shown that mercury is a peculiar stimulant of neural LINE-1 transposition, showing that there may be a link between environmental exposure to pollution and long-term genetic alterations in humans (Habibi et al., 2014). The potential of TEs to disrupt gene regulatory networks can be configured in this context as an evolutionary strategy for populations to “explore” their genetic capacity more rapidly under unfavorable conditions (Casacuberta and González, 2013). This has been recently studied in murine models, where experiments showcased how brain activity is particularly impacted: in rodents exposed to maternal immune activation or enriched environments, specific types of TEs exhibit altered expression in stress-sensitive brain regions, indicating that early-life environmental inputs can leave a molecular footprint on the brain (Richter et al., 2023); similarly, LINE-1 retrotransposons show region-specific activation in adult mouse brains in response to stress, suggesting a link between environmental adversity and neural vulnerability mediated by TE dynamics (Cappucci et al., 2018). These findings are also supported by studies highlighting how the eukaryotic stress response can influence the whole transposome, determining whether TEs remain quiescent or become transcriptionally active (Hunter et al., 2015). As such, TEs act as biosensors of environmental adversity, modulating gene expression and influencing development and behavior (Horváth et al., 2017). The epigenetic regulation of TEs under stress conditions further highlights their role in bridging short-term phenotypic responses and long-term genetic adaptation (Figure 2): from an evolutionary perspective, stress-induced TE activation provides a mechanism for introducing genetic variability at a pace much faster than traditional point mutations, especially in eukaryotic genomes that are otherwise constrained in mutational flexibility (Miousse et al., 2015). This capacity is particularly relevant in the context of neurodevelopment, where both physical and psychological insults during critical periods can have lasting genomic consequences. In humans, for example, steroid receptor signaling pathways and epigenetic modifications have been involved in stress-induced behavioural, psychiatric and mood disorders (Jiang et al., 2019; Mourtzi et al., 2021), and TE modulation of the respective regulatory networks could introduce another layer of variability and permanence to these phenotypes (Guffanti et al., 2014; Misiak et al., 2019; DeRosa et al., 2022). The hypothesis that TEs help bridge the gap between environmental adversity and neuropsychiatric ailments also offers a framework for understanding how stress can reshape the genome in ways that affect brain function and individual behavior across generations through heritable modifications (DeRosa et al., 2022).

#### Human pigmentation and tail-loss evolution

During the origin of the great apes *Hominidae*a lineage, approximately 14 million years ago, several phenotypic innovations occurred, from the enlarged brain volume and improved cognitive skills (Smaers et al., 2017; Friederici et al., 2024), to increased body size (Smith and Jungers, 1997) and diversification of diet (Andrews and Johnson, 2020). Another relevant phenotypic innovation is the loss of tail in anthropomorphic apes, which is proposed to have encouraged the rise of human bipedalism [reviewed in (Crompton et al., 2023)]. These “new” characteristics have been evolutionarily influenced - if not caused - by structural variants, in particular TEs (Zhou et al., 2023). In their study, Zhou and colleagues identify almost 16 thousand great ape-specific SVs, showing that most TE-related genes are involved in development and morphogenesis (Zhou et al., 2023). More recently, Xia and colleagues (Xia et al., 2024) discovered that the loss of tail in *Hominidae* is directly related to the presence of an intronic Hominoid-specific *AluY* in the *TBXT* gene (T-Box Transcription Factor T) on chromosome 6, which is reverse-oriented with respect to another intronic *Alu*. Their interaction promotes alternative splicing of *TBXT* by removing the sixth exon of this gene via the formation of a RNA hairpin structure and, in the end, leads to the Hominoid-specific tail-loss phenotype (Xia et al., 2024) (Figure 2).

Another example of TEs contributing to species- or population-specific characteristics is the evolution of human pigmentation: in this case, Authors showed how changes in *ASIP*’s (Agouti SIgnaling Protein) expression are due to the presence of two human-specific SVAs: the first SVA-F insertion led to an increase in skin pigmentation, caused by the reduced expression of the gene, in the ancestors of Neanderthals, Denisovans and modern humans, and is now *fixed* in *Homo sapiens* (Kamitaki et al., 2024) - meaning that the insertion is present in every individual and in every population. On the contrary, the insertion of a second, more recent SVA-F_1_ led to a decrease of pigmentation - and therefore a whiter skin - via higher gene expression in the ancestral populations of northwest Europeans (Kamitaki et al., 2024) (Figure 2).

#### Domestication and co-option of TE proteins and sequences

A further mechanism of integration and co-existence of TEs with their host genomes is represented by *domestication* of TE-derived sequences. In their work, Jangam and colleagues (Jangam et al., 2017) highlight three possible ways by which TE proteins (i.e., functional proteins synthesized from TE sequences) have been domesticated and co-opted as a response to the genetic conflicts between TEs and their hosts: 1) TE proteins co-option to repress TEs and retroviruses activity; 2) transition from actively transposing elements toward domestication due to TEs invasive strategies; 3) co-option of both TE proteins and sequences for novel biological processes (Jangam et al., 2017).

One of the most compelling examples of TE protein domestication is the emergence of the V(D)J system of acquired immunity in jawed vertebrates (Kapitonov and Jurka, 2005; Koonin and Krupovic, 2015; Huang et al., 2016), which include humans. Two major players of the V(D)J system are *RAG1* and *RAG2* proteins (Figure 2): the first coming directly from a transposase - an enzyme capable of cutting and splicing DNA - and the second being a descendant of an ancestral transposon (Jangam et al., 2017).

Another example of TE sequences domestication is that of PiggyBac Transposable Element Derived 5 (*PGBD5*), an evolutionarily conserved vertebrate DNA transposase-derived gene (Zapater et al., 2026). In their study, Zapater and colleagues show that *PGBD5* contributes to normal brain development in mice and humans, where its deficiency causes disorder of intellectual disability, movement and seizures. *PGBD5* may be required for normal nervous system development by mediating somatic DNA rearrangements in developing neurons: it is indeed expressed mostly in the brain, in both humans and mice (Zapater et al., 2026) (Figure 2).

A further case study is the co-option of SVA elements in human pluripotent stem cells: among the ∼3,700 copies of SVAs annotated in the human genome, Barnada (Barnada et al., 2022) reported that 751 are: 1) co-opted as functional *cis*-regulatory elements; 2) significantly closer to genes; and 3) harbor more TFBSs than non-co-opted SVAs (Barnada et al., 2022) (Figure 2). The Authors also show how SVA repression resulted in ∼3,000 differentially expressed genes, therefore modulating human gene expression (Barnada et al., 2022).

A recent perspective article also argues that KRAB zinc-finger proteins transformed the evolutionary problem of controlling transposable elements through TE-derived DNA regulation in a cell- and context-specific manner: KZFPs have helped domesticate mobile genetic elements into components of mammalian gene-regulatory networks, with consequences for development and disease (Davis et al., 2026).

Interestingly, TEs can act not only in *cis*, but also in *trans*, even if *trans* effects are more challenging to study: one of the most relevant examples of this action is the co-option of ERV and SINE sequences into binding sites for topological regulators of chromatin, for example, CTCF (CCCTC-binding factor) (Figure 2), which helped shaping and evolving chromatin looping and gene expression in Mammals (Bourque et al., 2008; Kunarso et al., 2010; Almeida et al., 2022).

These examples illustrate an important distinction in evaluating the evolutionary role of TEs. In some cases, the evidence extends beyond TE presence or association and supports a mechanistic link between a TE insertion and a phenotypic trait; in others, TE enrichment or differential distribution provides only a hypothesis for functional involvement. Therefore, a major challenge for the field is to move from identifying TE variants associated with evolutionary differences to experimentally demonstrating their effects on gene regulation, cellular phenotypes and fitness. This distinction is particularly important when considering complex traits, for which TE-mediated effects are likely to interact with multiple genetic and environmental factors.

## Transposable elements as genetic variability markers

Transposable elements mobilization in germline cells can enhance diversity through the production of potentially inheritable insertions, thereby generating human-specific polymorphisms. For instance, the analysis of the 1000 Genomes Project (1KGP) dataset (2,504 unrelated individuals from 26 different modern populations) identified ∼16,000 polymorphic TE loci (Gardner et al., 2017), 93% of which show a worldwide allele frequency <5%, indicating that overall polymorphic TEs are deleterious and have faced purifying selection (Rishishwar et al., 2018), but also showing promising results as valuable genetic markers for studies on human ancestry and evolution (Modenini et al., 2024).

In spite of being frequently under strong negative selection, the presence of polymorphisms in mobile element loci is an exciting potential source of information, in particular when analyzing modern human diversity, as polymorphic TEs have been shown to provide several advantages when compared to other more widely used genetic markers (Cordaux and Batzer, 2009; Ray and Batzer, 2011; Rishishwar et al., 2017). Firstly, polymorphic TEs shared among individuals are usually an example of identity by descent (IBD) (Batzer and Deininger, 2002), that is when two or more individuals share genetic segments because they inherited them from a common ancestor. Indeed, the high number of potential insertion sites in the genome and the low transposition rates mean that the probability of independent events of insertion in the same genomic location in two unrelated individuals is negligible: the most recent pedigree-based estimations calculated one in 40 births new *Alu* insertion and one new insertion in 63 births for LINE-1 and SVA (Feusier et al., 2019), even if previous studies based on comparative genomics and phylogenetics calculated a transposition rate for SVAs to be ∼1:900 births (Xing et al., 2009). The second reason is that newly inserted TEs rarely undergo deletion and, even when they do, they leave a molecular signature on the genome, making them highly stable polymorphisms (Rishishwar et al., 2015). Therefore, polymorphic TE markers are usually free of homoplasies (i.e., identical states that do not represent shared ancestry) (Ray et al., 2006), providing a more accurate indication of relationship with respect to classical genetic markers, such as single nucleotide polymorphisms (SNPs) or microsatellites (Ray and Batzer, 2011). Another advantage of using polymorphic TEs as variability markers is that the ancestral state of every locus can be defined as the *absence* of the insertion (Perna et al., 1992), which allows to draw population relationship trees with more confidence and without the necessity of a reference sequence to define ancestral/derived states. Finally, polymorphic TEs proved to be practically useful markers since they can be rapidly and accurately typed with PCR-based assays (Rishishwar et al., 2015).

Rishishwar’s (Rishishwar et al., 2015) and Gardner’s works (Gardner et al., 2017) were reinforced more recently by that of Watkins and colleagues (Watkins et al., 2020), who used the same approach on the Simons Genome Diversity Project (SGDP), identifying new polymorphic TEs not reported by the 1KGP and confirming that these objects are indeed exceptionally useful as variability and ancestry markers for human populations, while also potentially providing a basis for the study of medical susceptibilities [reviewed in (Kazazian and Moran, 2017; Ahmadi et al., 2020; Chénais, 2022)].

The emergence of evolutionary and ancestry-based medicine represents a new step towards precise healthcare and pharmacology, with great prospects for a better understanding of diseases and improved treatment outcomes (Ashley, 2016). The research focus has changed from the “one-size fits all” approach - and therefore, from generalized medicine - to understanding the influence of patterns in human genetic variation on disease pathology and treatment (Krainc and Fuentes, 2022). This new approach seeks to evaluate and consider differences among populations and even among individuals of the same group, in order to develop tailored therapies that optimize treatment efficacy (Krainc and Fuentes, 2022). Indeed, fine-scale population structure can predict complex disease risk within groups more reliably than what self-reported ethnicity has shown (Tatonetti and Elhadad, 2021). As we already discussed, the vast majority of TEs have been proven to be under purifying selection and mostly deleterious (Rishishwar et al., 2018): therefore, their inclusion in ancestry- and evolutionary-oriented genomic studies could be useful to understand their role in health and disease and integrated studies on TEs and other more popular genetic markers could unveil human evolutionary patterns that possibly contributed to several diseases, from cancer (Arora et al., 2025) to psychiatric disorders (Nesse, 2023; Jajoo et al., 2025).

## From genotype to phenotype: the brain as a case study

### Transposable elements in psychiatric disorders

The key part of brain evolution is changes in brain development, and there is significant evidence that TE-related mechanisms participated in shaping mammalian embryonic development, including neuronal differentiation (Ferrari et al., 2021) (Figure 3). Indeed, “both LTR and non-LTR retrotransposons appear to have contributed to mammalian brain evolution by acting as sources of novel non-coding RNAs, proteins, enhancers, RNA regulatory sites and sites for 3D genome organization” (Ferrari et al., 2021).

**FIGURE 3:**
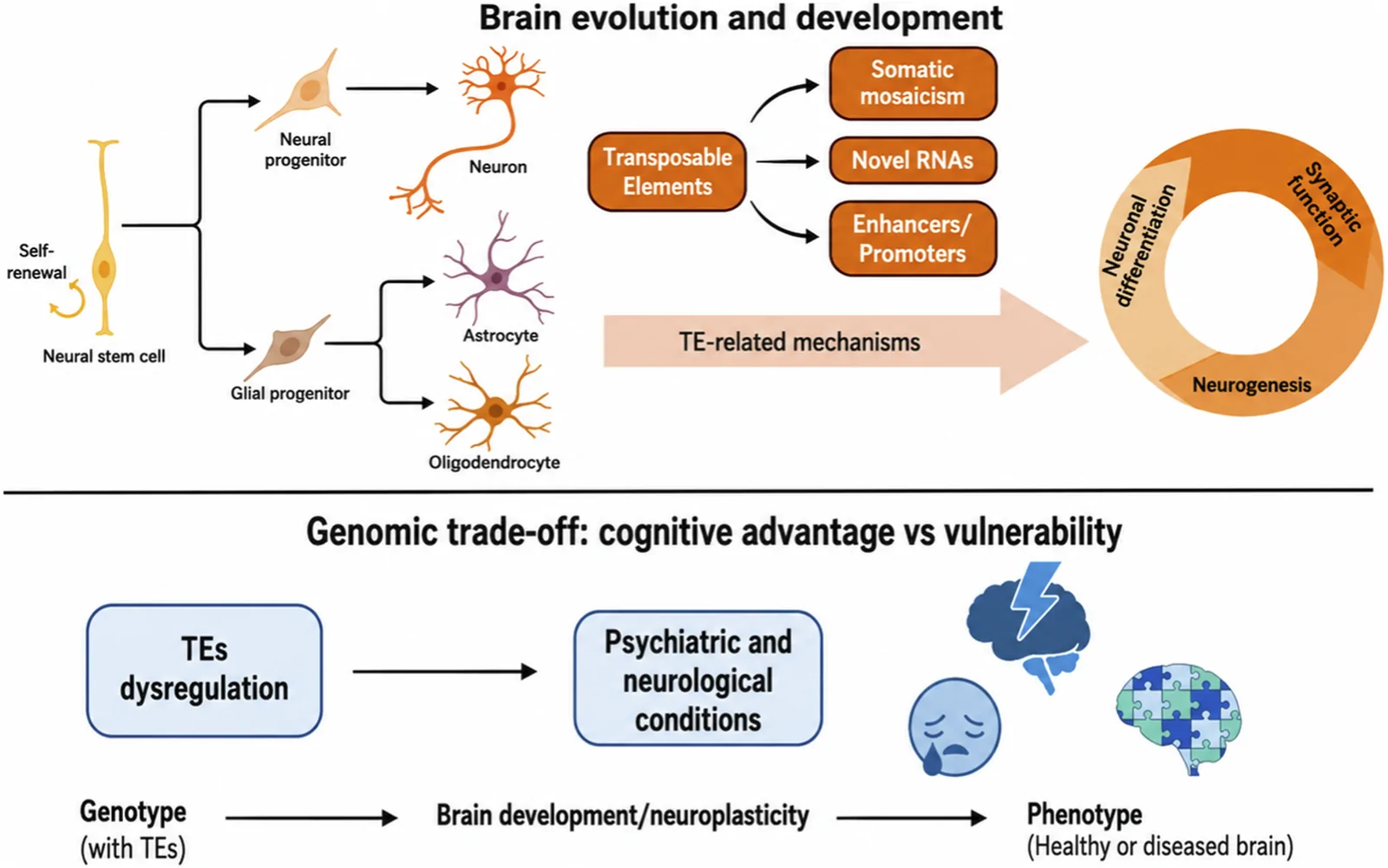
Mobile elements are both positive and detrimental players in the human brain. TEs mobilization could be the key for understanding psychiatric and neurological disorders, even if TEs are also involved in neuroplasticity, somatic mosaicism and, possibly, human brain evolution. Created with biorender.com.

On the other hand, recent data provide evidence for dysregulation of TEs in some neurological disorders (Cordaux and Batzer, 2009). Although the mechanistic details of the functional and evolutionary impact of TEs in the brain and nervous system are still unknown, a growing number of studies suggest that TEs contribute to neurodevelopmental/psychiatric disorders, including schizophrenia (Erwin et al., 2014; Guffanti et al., 2014; 2018; Erady et al., 2022; Ahn et al., 2023; Modenini et al., 2023), bipolar disorder (Erady et al., 2022; Ahn et al., 2023) and autism spectrum disorder (Okay et al., 2023; Saeliw et al., 2023; Spirito et al., 2023; Techaniyom et al., 2025) (Figure 3). These findings have major implications for understanding the neuroplasticity of the brain, which probably had a remarkable impact on brain evolution in primates, especially in *Hominids*, and could contribute to vulnerability to neurological disorders (Ahmadi et al., 2020).

HERVs are another family of TEs that have been frequently studied in neuropsychiatric diseases. In the last 2 decades, there has been mounting evidence that K and W families of Human Endogenous Retroviruses (HERV-K and HERV-W) are implicated in psychiatric disorders: for instance, abnormal expression of HERV-W and HERV-K has been found in blood, cerebrospinal fluid and post-mortem brain samples from patients diagnosed with schizophrenia, both at onset and in later stages of the disease (Christensen, 2016). HERV activity has also been investigated in other psychiatric conditions, such as bipolar disorder, major depression, autism spectrum disorder (ASD) and attention deficit hyperactivity disorder (ADHD) (Balestrieri et al., 2012; Guffanti et al., 2014; Duarte et al., 2024; Rangel et al., 2024).

LINEs and SINEs have also received attention for neurological disorders studies. LINEs have mostly been studied with reference to early and later brain development: Baillie (Baillie et al., 2011) found that protein-coding loci are disproportionately affected by non-LTR retrotransposons, with overrepresentation of LINE-1s in introns and *Alus* in exons. Overall, retrotransposon insertions seem to predominantly affect neurogenesis and synaptic function. Abrusán (Abrusán et al., 2013) proposed that a reduced expression of genes affected by LINE-1 insertions could potentially influence the biosynthesis and metabolism of different neurotransmitters such as dopamine, serotonin and glutamate.

The discovery of active TEs in the brain has raised questions about their influence on brain development and functioning (Reilly et al., 2013): on one hand, TE insertions may exert a controlling influence on flanking genes, causing a functionally relevant impact on the diversification of neuronal cell types or on the function of differentiated neurons. On the other hand, a detrimental impact of unregulated transposon expression must be considered: for instance, altered retrotransposon expression or function appear to be associated with neurodegeneration and aging [reviewed in (Ochoa Thomas et al., 2020; Ravel-Godreuil et al., 2021; Peze-Heidsieck et al., 2022; Copley and Shorter, 2023)].

Therefore, transposable elements seem to have shaped mammalian brain evolution and have been identified as key players in brain diseases. Therefore, in this context, the identification of TEs that could have contributed to *Homo sapiens* specific brain features is important to unveil the functional role that these elements had - and possibly still have - on human brain evolution.

### Neuroplasticity and brain evolution as byproducts of transposons mobility

Neuroplasticity is the brain’s capacity to reorganize itself by forming new neural connections and it was once believed to occur only during embryonic development; however, it continues throughout the lifespan, supporting learning, memory, and recovery from injury or disease (Gazerani, 2025).

Proper functioning of the nervous system depends on the establishment of a diverse repertoire of neuronal subtypes and the integration of individual cells into unique neuronal circuits (Figure 3). Within a neuronal subtype, cells display numerous phenotypes and differ in their molecular characteristics, firing patterns and connections (Erwin et al., 2014). A combination of several molecular mechanisms, including epigenetic regulation, alternative splicing and post-translational modifications, contribute to the generation of this neuronal diversity (Erwin et al., 2014).

In this context, Manolio and colleagues were among the first to suggest that somatic retrotransposition could help understand the unexplained variation seen in many psychiatric disorders that has evaded detection by conventional methods, such as genome-wide association studies (GWAS) (Manolio et al., 2009). This fundamental discrepancy, often referred to as the “missing heritability problem in psychiatry”, reveals the persistent gap between heritability estimates derived from family studies and the variance explained by identified genetic variants (Owen and Williams, 2021). Indeed, several psychiatric disorders have been known to run in families; however, GWAS-derived SNP variability accounts for only a fraction of the total predicted heritability (Van Calker and Serchov, 2021). One of the explanations could be that structural variants and transposable elements are not captured in standard genotyping arrays, but may contribute significantly to genetic risk (Modenini et al., 2023). In addition to this, epistatic and gene-environment interactions may play crucial roles in shaping psychiatric phenotypes, but these instances are difficult to model (Gillett, 2024), especially considering the polygenic nature of psychiatric disorders and the effect of epigenetic modification on TE mobilization (Bi et al., 2025; Yang et al., 2025).

To understand the *missing heritability problem,* is crucial to add that TEs have been also identified as responsible for somatic “mosaicism” (the presence of cells with distinct genotypes within one individual) (Muotri et al., 2005), which adds another level of complexity by generating genetic diversity across neurons (Figure 3). In particular, TEs increase their activity specifically during the differentiation of a neural precursor into a neuron (Muotri et al., 2005).

Furthermore, *genomic trade-off*, a mechanism by which “changes in the genome that are overall beneficial persist even though they also produce disease in a subset of individuals” (Sikela and Searles Quick, 2018), must also be considered when studying the impact of TEs on the human brain (Figure 3). This evolutionary mechanism is possibly ascribable to schizophrenia and ASD, the first being a real biological and evolutionary paradox: schizophrenia is indeed characterized by high heritability, but it is also associated with decreased reproductive success (Sikela and Searles Quick, 2018). In this context, scientists proposed several hypotheses to explain the emergence and persistence of psychiatric disorders in humans: for instance, the *price-to-pay* hypothesis (Crow, 1997; 2000) argues that “the primate brain, with the development of the neocortex, has become a more complex system enabling new competencies such as social cognition or language skills” (Sandroni and Chaumette, 2025) and that this increased brain complexity made the brain itself more vulnerable to dysfunctions.

Studying hippocampal intermediate progenitor cells (hpIPCs) of humans and chimpanzees, Patoori demonstrated that ∼2,500 genes are differentially expressed between humans and chimpanzee hpIPCs and that species-specific transposon-derived enhancers contribute to these transcriptomic differences (Patoori et al., 2022). Interestingly, species-specific enhancers are enriched for evolutionarily recent TEs, especially HERVs and SVAs, the last being pervasive regulators of hippocampal neurogenesis (Patoori et al., 2022). Moreover, as suggested by different works (Pontis et al., 2019; Turelli et al., 2020; Playfoot et al., 2021), successful neurogenesis depends partially on the repression of SVA elements during precise neuronal developmental stages, similarly to what it was previously discussed about HERVs. Therefore, Patoori and colleagues propose that hippocampal neuron development has been profoundly affected by the domestication of young TEs, which have been co-opted to serve as functional enhancers and promoters of the developing brain (Patoori et al., 2022). Similarly, a recent work suggested that L1 expression in hiPSCs is largely restricted to human-specific and hominoid-specific L1 elements, namely, L1HS, L1PA2 and L1PA3: expression of these elements correlates with lower levels of DNA methylation, therefore indicating that TEs can escape host’s repression mechanisms (Adami et al., 2025). The Authors highlight that LINE-1s provide one of the layers of hominoid-specific transcriptional complexity, especially in human pluripotent stem cells, and that this feature is important for the exit of pluripotency and early brain development (Adami et al., 2025).

Therefore, it is possible that structural variants such as TEs could take part in the aforementioned conditions, but at the same time could have contributed to the rise of human-specific cognitive abilities: indeed, as highlighted by Linker, some major physiological innovations in primate brain evolution temporally coincide with the appearance/expansion of primate-specific TEs (Linker et al., 2017). Another recent work suggests that a L1-derived lncRNA human-specific transcript, expressed exclusively during brain development, influences brain size: in fact, silencing of this L1-derived lncRNA results in reduced size of cerebral organoids and premature differentiation of neural progenitors (Garza et al., 2023). Moreover, a recent work defined TEs, in particular LINE-1s, “as both *architects* of neuronal diversity and *saboteurs* of genomic integrity” (Izquierdo-Useros et al., 2026): the Authors point out that it is fundamental to integrate the current views of TEs as both evolutionary catalysts and pathological triggers, in order to understand their real impact on human brain functions, evolution and diseases. Interestingly, in recent works, TEs emerged as related to behavioral phenotypes, such as the regulation of both positive and negative emotions [reviewed in (Gaudi et al., 2016)]: the Authors conclude that TEs could be key players in shaping the dynamic neural circuitry processes underlying emotional regulation.

For the study of human cognitive evolution, a central priority is therefore to establish causal links between TE-derived variation and neural phenotypes. Comparative analyses across primates and archaic and modern human genomes, particularly when combined with long-read sequencing and cell-type-specific genomic data, can help identify TE insertions that are both evolutionarily novel and functionally active in the developing brain. These candidates could then be tested experimentally using perturbation approaches in relevant cellular and organoid models to determine whether they alter gene regulation, neuronal differentiation, connectivity or other measurable neural phenotypes. Integrating these functional experiments with population genetic and evolutionary analyses will be essential to distinguish TE variants that merely accompany evolutionary change from those that contributed directly to it. Ancient DNA represents an especially valuable component of this strategy because it can provide a temporal framework for reconstructing the emergence and spread of candidate TE variants, however methodological improvements are still required to reliably detect TEs in degraded ancient genomes. Although dysregulated TE activity has also been investigated in neurodevelopmental and psychiatric disorders, these observations should be considered here primarily as evidence for the broader biological consequences of TE activity in the nervous system rather than as a separate evolutionary framework. The key evolutionary question is whether the same TE-mediated mechanisms that contribute to neuronal diversity and developmental plasticity have also been recruited during human and primate evolution to generate functionally relevant changes in brain biology. At present, evidence for such a connection remains suggestive rather than conclusive.

## Towards a synthesis: transposable elements as functional innovators

In the previous paragraphs we showed how the view on transposable elements evolved since their discovery by Barbara McClintock. That was exceptional work: scientists did not even know which was the DNA structure, but she discovered that some “mutable loci” existed in maize.

Later on, the discovery of transposons as variants that contributed to shaping some phenotypes identified them as “mobile bricks” of regulation and innovation, therefore participating in human genome’s evolution. Therefore, TEs can be seen as something slightly different from just being a “background noise” for their host genomes, as we detailed their role of adaptive flexibility instruments *used* by the hosts themselves, even if we must point out that, in principle, TEs could not be generally considered beneficial, given their parasitic nature.

Comparative genomics and long-read WGS now allow for a detailed analysis of TE insertion sequences and polymorphisms, as well as for distinguishing between *conservation* and *innovation* when studying mobile elements. A high number of TE-derived sequences have been identified as conserved among different *taxa*, highlighting their functional and regulatory impact on the host genome and their valuable contribution to genome evolution, in terms of genetic variability and flexibility. Moreover, differences in TE content among *taxa* and populations of the same species allowed for studies on differentiation, local adaptation and phenotypic innovations.

Furthermore, TEs have been implicated in brain plasticity and evolution, especially when looking at primates and *Homo*; TEs could explain the so-called “evolutionary paradox” and “missing heritability problem” of psychiatric diseases, as classic approaches to study the genetic bases of these conditions did not entirely account for the variation observed among affected individuals.

In this context, a non-negligible number of tools and softwares to capture TE diversity, impact, regulatory functions, etc., have been the focus of intense research in the last decades. The recent availability of a considerable amount of aDNA sequences could have contributed to a new approach to study the TE-driven evolution of humans. However, problems when dealing with this type of sequences - for example, the high rates of degradation and base pair substitutions - led to a missing point in molecular anthropology.

Across the multidisciplinary evidence explored in this review, a unifying picture emerges: transposable elements are not only mere genomic “parasites” or neutral passengers, but they also have consistently operated as functional innovators in human evolution. Indeed, the cumulative information presented here, from the adaptive landscapes of high-altitude populations to neurogenomic responses under environmental stress, strongly suggest that TEs are not just tolerated, but exploited by the human genome. This view is increasingly supported by the observation that TE activity intersects functionally with core biological processes, modifying the regulatory framework of the human genome, creating new promoters, enhancers, transcription factor binding sites and alternative splicing motifs. Whether embedded in structural variants underlying pigmentation differences or reshaping brain gene expression in response to developmental stress, TEs frequently exert their influence by repurposing existing genetic circuitry in a non-random fashion: certain TEs are preferentially retained, epigenetically regulated or domesticated when their functions favour host fitness, pointing to the emergence of selective co-option over evolutionary time.

In this context, a major challenge for future research will be to determine when TE-derived sequences represent genuine drivers of evolutionary change rather than genomic by-products of it. Although increasing evidence indicates that TEs can contribute to regulatory elements, transcriptional networks, structural variation and, in some cases, phenotypic traits, the functional consequences of many TE insertions remain difficult to establish. In particular, the conservation or differential abundance of a TE does not by itself demonstrate positive selection or a causal contribution to a phenotype.

Nevertheless, from a mechanistic standpoint, TEs have contributed functional novelty at three main levels: sequence, structure, and expression control. At the sequence level, TEs generate novel genetic variability, introducing potential protein-coding changes through large-scale rearrangements, transductions and deletions. More interestingly, however, TEs act as expression modifiers, inserting regulatory modules that become conditionally active and define gene expression with tissue specificity and temporal precision. One compelling example is the role of TEs as stress-responsive regulators. As discussed, both external and internal stimuli can transiently activate TEs, positioning them as evolutionary accelerants that introduce environmentally-induced phenotypic plasticity into the genomic substrate and imprint lasting regulatory modifications. Such changes, once written in the genome, are potentially heritable, creating a bridge between epigenetic responsiveness and long-term evolutionary outcomes. Moreover, the existence of TE-derived proteins and functional sequences provides examples of molecular exaptation, whereby formerly selfish elements are co-opted into useful cellular roles: their molecular repurposing highlights the creative potential of TEs as genomic “raw material”. The binary between “junk” and “functional” DNA is therefore dismantled, replacing it with a continuum between latency and functionality. Methodologically, a more complete understanding of TE-mediated evolution will therefore require the integration of comparative genomics, population genetics and functional approaches capable of distinguishing correlation from causation.

## Conclusion

In light of these findings, we believe that TEs should be reframed not merely as mutational burden or “background noise”, but also as genome-encoded mechanisms of innovation. Their replicative nature, stress responsiveness and epigenetic regulation equip them with the ability to generate complexity, plasticity and heritability very quickly. They serve as reservoirs of regulatory potential and accelerators of evolutionary change, particularly in systems like neurodevelopment, immunity, and environmental adaptation where speed and diversity are paramount for survival and fitness. Understanding the contributions of TEs to human evolution requires the acknowledgement that genomes have never been static blueprints underlying simple phenotypic manifestations, but are regulated ecosystems capable of fast and radical innovation.

An evolutionary perspective on TEs also provides a concrete framework for identifying which TE activities are functionally relevant to disease. Rather than considering TE activation or insertion as a largely undifferentiated source of genomic instability, future studies should ask whether specific TE variants have been preferentially retained, expanded, or co-opted in particular cellular or disease contexts, and whether these events confer a measurable phenotypic advantage. This question is particularly well suited to emerging pangenomic and long-read sequencing approaches. The current human pangenome already demonstrates that a portion of human genetic diversity is represented by structural variation that is poorly captured by a single linear reference genome, with pangenomic analyses revealing millions of bases of previously underrepresented sequence and substantially increasing structural variant discovery relative to the GRCh38 linear human reference (Liao et al., 2023). Because many TE loci are repetitive and poorly mappable with short reads, extending pangenomic analyses specifically to TE insertions, deletions, copy-number changes and their internal sequence variation should help in distinguishing population-specific and rare TE alleles and in determining whether particular TE configurations (i.e., presence/absence, sequence alterations, etc.) are enriched in disease-associated phenotypes. Long-read sequencing has already revealed extensive structural variation within SVA elements at neurological disease-associated loci, including *BIN1* and *CD2AP* in Alzheimer’s disease and *BCKDK* in Parkinson’s disease, revealing TE sequence variation as a hidden layer of genetic change underlying disease risk (Van Bree et al., 2022). These observations provide a testable evolutionary hypothesis: TE variants that alter disease-associated regulatory networks should not reflect neutral variation, but exhibit reproducible associations with gene expression and disease phenotypes across genetically diverse individuals.

Long-read sequencing will be particularly important for testing this hypothesis because it can resolve individual TE copies within their genomic context: PacBio HiFi sequencing provides accurate long reads suitable for resolving complex insertions and haplotypes, whereas Oxford Nanopore Technologies (ONT) sequencing can generate very long reads and directly detect DNA modifications such as methylation (Chuong et al., 2017; Liu and Conesa, 2025). Such approaches have been especially informative in the study of cancer, where TE activity can be highly cell-type- and context-dependent. For example, TE-derived regulatory sequences have been shown to function as tissue-specific enhancers in colorectal and liver cancers (Karttunen et al., 2023), while large-scale analyses have identified recurrent TE-derived promoter activation events associated with oncogene expression across multiple cancer types (Jang et al., 2019). Similarly, somatic TE insertions have been identified in tumors, impacting the cell regulatory landscape and disrupting gene expression (Diaz-Portal et al., 2026).

The evolutionary framework can likewise guide investigation of TE-mediated regulatory mechanisms beyond their ability to transpose. TE-derived sequences contain combinations of transcription-factor binding sites that can be repeatedly co-opted as promoters, enhancers or insulators. Consequently, an important future direction will be to define whether TE transcriptional activation creates a novel regulatory state that is advantageous or detrimental to a particular disease cell population. This could be addressed by integrating multimodal analyses (for example, a combination of long-read sequencing, transcriptomics and methylomics) that would allow to test whether TE activation precedes changes in gene regulation during disease progression, or whether particular TE subfamilies are repeatedly recruited by the same transcription factors. In cancer, this approach is particularly compelling because TE reactivation can have opposite consequences: TE-derived regulatory elements can promote oncogene expression, whereas TE-derived transcripts can also trigger innate immune responses through “viral mimicry” (Roulois et al., 2015; Jang et al., 2019).

These developments highlight the need for computational approaches designed around the evolutionary properties of repetitive DNA, rather than treating TEs as problematic regions to be filtered out. A large-scale integration of graph-based pangenomic references, haplotype-resolved assemblies and improved TE-aware variant callers may be the foundation for approaches capable of linking TE sequence and epigenetic features to disease phenotypes. Importantly, such analyses should distinguish between TE family-level activities and locus-specific effects, since individual copies within the same TE family can have very different evolutionary ages, sequence compositions, epigenetic states and regulatory functions. The required upgrade in this field is to shift from descriptive measurements of TE activity to predictive models that identify which TE loci are likely to influence disease and why. In this sense, an evolutionary lens generates specific hypotheses around TEs that can carry the potential to reveal genetic and regulatory variation invisible to conventional reference-based analyses. In turn, this may allow for the identification of new mechanisms, biomarkers and actionable vulnerabilities in disease.

## References

[B1] Abrusán G. Zhang Y. Szilágyi A. (2013). Structure prediction and analysis of DNA transposon and LINE retrotransposon proteins. J. Biol. Chem. 288, 16127–16138. 10.1074/jbc.M113.451500 23530042 PMC3668768

[B2] Adami A. Garza R. Gerdes P. Johansson P. A. Dorazehi F. Koutounidou S. (2025). LINE-1 retrotransposons mediate cis-acting transcriptional control in human pluripotent stem cells and regulate early brain development. Cell Genomics 5, 100979. 10.1016/j.xgen.2025.100979 PMC12791003 40848716

[B3] Ahmadi A. De Toma I. Vilor-Tejedor N. Eftekhariyan Ghamsari M. R. Sadeghi I. (2020). Transposable elements in brain health and disease. Ageing Res. Rev. 64, 101153. 10.1016/j.arr.2020.101153 32977057

[B4] Ahn H. W. Worman Z. F. Lechsinska A. Payer L. M. Wang T. Malik N. (2023). Retrotransposon insertions associated with risk of neurologic and psychiatric diseases. EMBO Rep. 24, e55197. 10.15252/embr.202255197 36367221 PMC9827563

[B5] Almeida M. V. Vernaz G. Putman A. L. K. Miska E. A. (2022). Taming transposable elements in vertebrates: from epigenetic silencing to domestication. Trends Genet. 38, 529–553. 10.1016/j.tig.2022.02.009 35307201

[B6] Andrews P. Johnson R. J. (2020). Evolutionary basis for the human diet: consequences for human health. J. Intern Med. 287, 226–237. 10.1111/joim.13011 31733113

[B7] Andrews G. Fan K. Pratt H. E. Phalke N. Zoonomia C. S Karlsson E. K. (2023). Mammalian evolution of human cis-regulatory elements and transcription factor binding sites. Science 380, eabn7930. 10.1126/science.abn7930 37104580

[B8] Arora K. Suehnholz S. P. Zhang H. Ostrovnaya I. Kundra R. Nandakumar S. (2025). Genetic ancestry–based differences in biomarker-based eligibility for precision oncology therapies. JAMA Oncol. 11, 310. 10.1001/jamaoncol.2024.5794 PMC11926650 39786754

[B9] Ashley E. A. (2016). Towards precision medicine. Nat. Rev. Genet. 17, 507–522. 10.1038/nrg.2016.86 27528417

[B10] Autio M. I. Bin Amin T. Perrin A. Wong J. Y. Foo R. S.-Y. Prabhakar S. (2021). Transposable elements that have recently been mobile in the human genome. BMC Genomics 22, 789. 10.1186/s12864-021-08085-0 PMC8567694 34732136

[B11] Babaian A. Mager D. L. (2016). Endogenous retroviral promoter exaptation in human cancer. Mob. DNA 7, 24. 10.1186/s13100-016-0080-x 27980689 PMC5134097

[B12] Baillie J. K. Barnett M. W. Upton K. R. Gerhardt D. J. Richmond T. A. De Sapio F. (2011). Somatic retrotransposition alters the genetic landscape of the human brain. Nature 479, 534–537. 10.1038/nature10531 22037309 PMC3224101

[B13] Balestrieri E. Arpino C. Matteucci C. Sorrentino R. Pica F. Alessandrelli R. (2012). HERVs expression in Autism Spectrum Disorders. PLoS One 7, e48831. 10.1371/journal.pone.0048831 23155411 PMC3498248

[B14] Bao W. Kojima K. K. Kohany O. (2015). Repbase Update, a database of repetitive elements in eukaryotic genomes. Mob. DNA 6, 11. 10.1186/s13100-015-0041-9 26045719 PMC4455052

[B15] Barnada S. M. Isopi A. Tejada-Martinez D. Goubert C. Patoori S. Pagliaroli L. (2022). Genomic features underlie the co-option of SVA transposons as cis-regulatory elements in human pluripotent stem cells. PLoS Genet. 18, e1010225. 10.1371/journal.pgen.1010225 PMC9239442 35704668

[B16] Batzer M. A. Deininger P. L. (2002). Alu repeats and human genomic diversity. Nat. Rev. Genet. 3, 370–379. 10.1038/nrg798 11988762

[B17] Beall C. M. Cavalleri G. L. Deng L. Elston R. C. Gao Y. Knight J. (2010). Natural selection on *EPAS1* (*HIF2α*) associated with low hemoglobin concentration in Tibetan highlanders. Proc. Natl. Acad. Sci. U.S.A. 107, 11459–11464. 10.1073/pnas.1002443107 20534544 PMC2895075

[B18] Bi H. Jin J. Sun M. Chen M. Li X. Wang Y. (2025). Epigenetic changes caused by early life stress in the pathogenesis of depression. Eur. J. Pharmacol. 999, 177671. 10.1016/j.ejphar.2025.177671 40288560

[B19] Bourque G. Leong B. Vega V. B. Chen X. Lee Y. L. Srinivasan K. G. (2008). Evolution of the mammalian transcription factor binding repertoire via transposable elements. Genome Res. 18, 1752–1762. 10.1101/gr.080663.108 18682548 PMC2577865

[B20] Bourque G. Burns K. H. Gehring M. Gorbunova V. Seluanov A. Hammell M. (2018). Ten things you should know about transposable elements. Genome Biol. 19, 199. 10.1186/s13059-018-1577-z 30454069 PMC6240941

[B21] Briggs A. W. Stenzel U. Johnson P. L. F. Green R. E. Kelso J. Prüfer K. (2007). Patterns of damage in genomic DNA sequences from a Neandertal. Proc. Natl. Acad. Sci. U. S. A. 104, 14616–14621. 10.1073/pnas.0704665104 17715061 PMC1976210

[B22] Cao X. Zhang Y. Payer L. M. Lords H. Steranka J. P. Burns K. H. (2020). Polymorphic mobile element insertions contribute to gene expression and alternative splicing in human tissues. Genome Biol. 21, 185. 10.1186/s13059-020-02101-4 32718348 PMC7385971

[B23] Cappucci U. Torromino G. Casale A. M. Camon J. Capitano F. Berloco M. (2018). Stress-induced strain and brain region-specific activation of LINE-1 transposons in adult mice. Stress 21, 575–579. 10.1080/10253890.2018.1485647 29996702

[B24] Casacuberta E. González J. (2013). The impact of transposable elements in environmental adaptation. Mol. Ecol. 22, 1503–1517. 10.1111/mec.12170 23293987

[B25] Cerruti Y. Fusco D. Jahanbin M. Marnetto D. Provero P. (2024). Sequence Composition and Conservation Predict the Phenotypic Relevance of Transposable Elements. 10.1101/2024.03.11.584453

[B26] Chen J. Basting P. J. Han S. Garfinkel D. J. Bergman C. M. (2023). Reproducible evaluation of transposable element detectors with McClintock 2 guides accurate inference of Ty insertion patterns in yeast. Mob. DNA 14, 8. 10.1186/s13100-023-00296-4 PMC10347736 37452430

[B27] Chénais B. (2022). Transposable elements and human diseases: mechanisms and implication in the response to environmental pollutants. Int. J. Mol. Sci. 23, 2551. 10.3390/ijms23052551 PMC8910135 35269693

[B28] Christensen T. (2016). Human endogenous retroviruses in neurologic disease. APMIS 124, 116–126. 10.1111/apm.12486 26818266

[B29] Chu C. Borges-Monroy R. Viswanadham V. V. Lee S. Li H. Lee E. A. (2021). Comprehensive identification of transposable element insertions using multiple sequencing technologies. Nat. Commun. 12, 3836. 10.1038/s41467-021-24041-8 PMC8219666 34158502

[B30] Chuong E. B. Elde N. C. Feschotte C. (2017). Regulatory activities of transposable elements: from conflicts to benefits. Nat. Rev. Genet. 18, 71–86. 10.1038/nrg.2016.139 27867194 PMC5498291

[B31] Copley K. E. Shorter J. (2023). Repetitive elements in aging and neurodegeneration. Trends Genet. 39, 381–400. 10.1016/j.tig.2023.02.008 PMC10121923 36935218

[B32] Cordaux R. Batzer M. A. (2009). The impact of retrotransposons on human genome evolution. Nat. Rev. Genet. 10, 691–703. 10.1038/nrg2640 19763152 PMC2884099

[B33] Crompton R. H. Sellers W. Davids K. McClymont J. (2023). Biomechanics and the origins of human bipedal walking: the last 50 years. J. Biomechanics 157, 111701. 10.1016/j.jbiomech.2023.111701 37451208

[B34] Crow T. J. (1997). Is schizophrenia the price that Homo sapiens pays for language? Schizophr. Res. 28, 127–141. 10.1016/s0920-9964(97)00110-2 9468348

[B35] Crow T. J. (2000). Schizophrenia as the price that homo sapiens pays for language: a resolution of the central paradox in the origin of the species. Brain Res. Brain Res. Rev. 31, 118–129. 10.1016/s0165-0173(99)00029-6 10719140

[B36] Davis J. Voicu D. Chitnavis U. Jaksina J. Imbeault M. (2026). The role of KRAB zinc-finger proteins in expanding the domestication potential of transposable elements. Nat. Genet. 58, 492–502. 10.1038/s41588-025-02498-3 41720928

[B37] Deininger P. (2011). Alu elements: know the SINEs. Genome Biol. 12, 236. 10.1186/gb-2011-12-12-236 22204421 PMC3334610

[B38] DeRosa H. Richter T. Wilkinson C. Hunter R. G. (2022). Bridging the gap between environmental adversity and neuropsychiatric disorders: the role of transposable elements. Front. Genet. 13, 813510. 10.3389/fgene.2022.813510 PMC9196244 35711940

[B39] Diaz-Portal L. Sotelo-Fonseca J. E. Rodriguez-Martin B. (2026). “Transposable elements in human cancer: regulation, activation, and genomic consequences,” in Advances in Genetics (Elsevier), 125–187. 10.1016/bs.adgen.2025.11.004 42219646

[B40] DiRusso J. A. Clark A. T. (2023). Transposable elements in early human embryo development and embryo models. Curr. Opin. Genet. and Dev. 81, 102086. 10.1016/j.gde.2023.102086 PMC10917458 37441874

[B41] Djebali S. Davis C. A. Merkel A. Dobin A. Lassmann T. Mortazavi A. (2012). Landscape of transcription in human cells. Nature 489, 101–108. 10.1038/nature11233 22955620 PMC3684276

[B42] Duarte R. R. R. Pain O. Bendall M. L. de Mulder Rougvie M. Marston J. L. Selvackadunco S. (2024). Integrating human endogenous retroviruses into transcriptome-wide association studies highlights novel risk factors for major psychiatric conditions. Nat. Commun. 15, 3803. 10.1038/s41467-024-48153-z PMC11111684 38778015

[B43] Erady C. Amin K. Onilogbo T. O. A. E. Tomasik J. Jukes-Jones R. Umrania Y. (2022). Novel open reading frames in human accelerated regions and transposable elements reveal new leads to understand schizophrenia and bipolar disorder. Mol. Psychiatry 27, 1455–1468. 10.1038/s41380-021-01405-6 PMC9095477 34937870

[B44] Erwin J. A. Marchetto M. C. Gage F. H. (2014). Mobile DNA elements in the generation of diversity and complexity in the brain. Nat. Rev. Neurosci. 15, 497–506. 10.1038/nrn3730 25005482 PMC4443810

[B45] Evrony G. D. Cai X. Lee E. Hills L. B. Elhosary P. C. Lehmann H. S. (2012). Single-neuron sequencing analysis of L1 retrotransposition and somatic mutation in the human brain. Cell 151, 483–496. 10.1016/j.cell.2012.09.035 23101622 PMC3567441

[B46] Ewing A. D. Smits N. Sanchez-Luque F. J. Faivre J. Brennan P. M. Richardson S. R. (2020). Nanopore sequencing enables comprehensive transposable element epigenomic profiling. Mol. Cell 80, 915–928.e5. 10.1016/j.molcel.2020.10.024 33186547

[B47] Faulkner G. J. Kimura Y. Daub C. O. Wani S. Plessy C. Irvine K. M. (2009). The regulated retrotransposon transcriptome of mammalian cells. Nat. Genet. 41, 563–571. 10.1038/ng.368 19377475

[B48] Fedoroff N. V. (2012). Transposable elements, epigenetics, and genome evolution. Science 338, 758–767. 10.1126/science.338.6108.758 23145453

[B49] Ferrari R. Grandi N. Tramontano E. Dieci G. (2021). Retrotransposons as drivers of Mammalian brain evolution. Life (Basel) 11, 376. 10.3390/life11050376 33922141 PMC8143547

[B50] Feschotte C. (2008). Transposable elements and the evolution of regulatory networks. Nat. Rev. Genet. 9, 397–405. 10.1038/nrg2337 18368054 PMC2596197

[B51] Feusier J. Watkins W. S. Thomas J. Farrell A. Witherspoon D. J. Baird L. (2019). Pedigree-based estimation of human mobile element retrotransposition rates. Genome Res. 29, 1567–1577. 10.1101/gr.247965.118 31575651 PMC6771411

[B52] Forcier T. Cheng E. Tam O. H. Wunderlich C. Castilla-Vallmanya L. Jones J. L. (2026). Tesingle Enables Locus-specific Transposable Element Expression Analysis at single-cell Resolution. 10.64898/2026.03.19.712984

[B53] Fort V. Khelifi G. Hussein S. M. I. (2021). Long non-coding RNAs and transposable elements: a functional relationship. Biochim. Biophys. Acta Mol. Cell Res. 1868, 118837. 10.1016/j.bbamcr.2020.118837 32882261

[B54] Friederici A. D. Wittig R. M. Anwander A. Eichner C. Gräßle T. Jäger C. (2024). Brain structure and function: a multidisciplinary pipeline to study hominoid brain evolution. Front. Integr. Neurosci. 17, 1299087. 10.3389/fnint.2023.1299087 PMC10800984 38260006

[B55] Friedli M. Trono D. (2015). The developmental control of transposable elements and the evolution of higher species. Annu. Rev. Cell Dev. Biol. 31, 429–451. 10.1146/annurev-cellbio-100814-125514 26393776

[B56] Gardner E. J. Lam V. K. Harris D. N. Chuang N. T. Scott E. C. Pittard W. S. (2017). The Mobile Element Locator Tool (MELT): population-scale mobile element discovery and biology. Genome Res. 27, 1916–1929. 10.1101/gr.218032.116 PMC5668948 28855259

[B57] Garza R. Atacho D. A. M. Adami A. Gerdes P. Vinod M. Hsieh P. (2023). LINE-1 retrotransposons drive human neuronal transcriptome complexity and functional diversification. Sci. Adv. 9, eadh9543. 10.1126/sciadv.adh9543 PMC10619931 37910626

[B58] Gasparotto E. Burattin F. V. Di Gioia V. Panepuccia M. Ranzani V. Marasca F. (2023). Transposable elements Co-Option in genome evolution and gene regulation. IJMS 24, 2610. 10.3390/ijms24032610 PMC9917352 36768929

[B59] Gaudi S. Guffanti G. Fallon J. Macciardi F. (2016). Epigenetic mechanisms and associated brain circuits in the regulation of positive emotions: a role for transposable elements. J Comp. Neurology 524, 2944–2954. 10.1002/cne.24046 27224878

[B60] Gazerani P. (2025). The neuroplastic brain: current breakthroughs and emerging frontiers. Brain Res. 1858, 149643. 10.1016/j.brainres.2025.149643 40280532

[B61] Gebrie A. (2023). Transposable elements as essential elements in the control of gene expression. Mob. DNA 14, 9. 10.1186/s13100-023-00297-3 PMC10439571 37596675

[B62] Gerdes P. Richardson S. R. Mager D. L. Faulkner G. J. (2016). Transposable elements in the mammalian embryo: pioneers surviving through stealth and service. Genome Biol. 17, 100. 10.1186/s13059-016-0965-5 27161170 PMC4862087

[B63] Gillett G. (2024). The problem with genetic heritability estimates in psychiatry: “missing heritability” or missed cross-cultural environmental variation? Psychiatry Res. 336, 115916. 10.1016/j.psychres.2024.115916 38640570

[B64] Groza C. Bourque G. Goubert C. (2023). A pangenome approach to detect and genotype TE insertion polymorphisms. Methods Mol. Biol. 2607, 85–94. 10.1007/978-1-0716-2883-6_5 36449159

[B65] Groza C. Chen X. Wheeler T. J. Bourque G. Goubert C. (2024). A unified framework to analyze transposable element insertion polymorphisms using graph genomes. Nat. Commun. 15, 8915. 10.1038/s41467-024-53294-2 39414821 PMC11484939

[B66] Gu X. Wang M. Zhang X.-O. (2024). TE-TSS: an integrated data resource of human and mouse transposable element (TE)-derived transcription start site (TSS). Nucleic Acids Res. 52, D322–D333. 10.1093/nar/gkad1048 37956335 PMC10767810

[B67] Guffanti G. Gaudi S. Fallon J. H. Sobell J. Potkin S. G. Pato C. (2014). Transposable elements and psychiatric disorders. Am. J. Med. Genet. B Neuropsychiatr. Genet. 165B, 201–216. 10.1002/ajmg.b.32225 24585726 PMC12848872

[B68] Guffanti G. Bartlett A. Klengel T. Klengel C. Hunter R. Glinsky G. (2018). Novel bioinformatics approach identifies transcriptional profiles of lineage-specific transposable elements at distinct loci in the human dorsolateral prefrontal cortex. Mol. Biol. Evol. 35, 2435–2453. 10.1093/molbev/msy143 30053206 PMC6188555

[B69] Guichard E. Peona V. Malagoli Tagliazucchi G. Abitante L. Jagoda E. Musella M. (2018). Impact of non-LTR retrotransposons in the differentiation and evolution of anatomically modern humans. Mob. DNA 9, 28. 10.1186/s13100-018-0133-4 30147753 PMC6094920

[B70] Habibi L. Shokrgozar M. A. Tabrizi M. Modarressi M. H. Akrami S. M. (2014). Mercury specifically induces LINE-1 activity in a human neuroblastoma cell line. Mutat. Research/Genetic Toxicol. Environ. Mutagen. 759, 9–20. 10.1016/j.mrgentox.2013.07.015 24240092

[B71] Halldorsson B. V. Eggertsson H. P. Moore K. H. S. Hauswedell H. Eiriksson O. Ulfarsson M. O. (2022). The sequences of 150,119 genomes in the UK Biobank. Nature 607, 732–740. 10.1038/s41586-022-04965-x 35859178 PMC9329122

[B72] Horváth V. Merenciano M. González J. (2017). Revisiting the relationship between transposable elements and the eukaryotic stress response. Trends Genet. 33, 832–841. 10.1016/j.tig.2017.08.007 28947157

[B73] Hoyt S. J. Storer J. M. Hartley G. A. Grady P. G. S. Gershman A. de Lima L. G. (2022). From telomere to telomere: the transcriptional and epigenetic state of human repeat elements. Science 376, eabk3112. 10.1126/science.abk3112 PMC9301658 35357925

[B74] Huang S. Tao X. Yuan S. Zhang Y. Li P. Beilinson H. A. (2016). Discovery of an active RAG Transposon illuminates the origins of V(D)J recombination. Cell 166, 102–114. 10.1016/j.cell.2016.05.032 27293192 PMC5017859

[B75] Hunter R. G. (2020). Stress, adaptation, and the deep genome: why transposons matter. Integr. Comp. Biol. 60, 1495–1505. 10.1093/icb/icaa050 32483621

[B76] Hunter R. G. Gagnidze K. McEwen B. S. Pfaff D. W. (2015). Stress and the dynamic genome: steroids, epigenetics, and the transposome. Proc. Natl. Acad. Sci. U.S.A. 112, 6828–6833. 10.1073/pnas.1411260111 25385609 PMC4460487

[B77] International Human Genome Sequencing Consortium (2004). Finishing the euchromatic sequence of the human genome. Nature 431, 931–945. 10.1038/nature03001 15496913

[B78] Izquierdo-Useros N. Dierssen M. Clotet B. Elizalde-Torrent A. Carriba P. (2026). “Retrotransposons as both ‘architects’ and ‘saboteurs’ in the nervous system,” in Advances in Genetics (Elsevier), 101–123. 10.1016/bs.adgen.2025.11.002 42219645

[B79] Jajoo A. Balakundi V. Chatzinakos C. Aruldass A. Wen C. Kolmans I. G. C. (2025). Leveraging genomic and transcriptomic data of diverse ancestry to uncover mechanisms of psychiatric risk in the adult and developing brain. Nat. Commun. 17, 1179. 10.1038/s41467-025-67941-9 41461652 PMC12858809

[B80] Jang H. S. Shah N. M. Du A. Y. Dailey Z. Z. Pehrsson E. C. Godoy P. M. (2019). Transposable elements drive widespread expression of oncogenes in human cancers. Nat. Genet. 51, 611–617. 10.1038/s41588-019-0373-3 30926969 PMC6443099

[B81] Jangam D. Feschotte C. Betrán E. (2017). Transposable element domestication as an adaptation to evolutionary conflicts. Trends Genet. 33, 817–831. 10.1016/j.tig.2017.07.011 28844698 PMC5659911

[B82] Jiang S. Postovit L. Cattaneo A. Binder E. B. Aitchison K. J. (2019). Epigenetic modifications in stress response genes associated with childhood trauma. Front. Psychiatry 10, 808. 10.3389/fpsyt.2019.00808 31780969 PMC6857662

[B83] Jin Y. Tam O. H. Paniagua E. Hammell M. (2015). TEtranscripts: a package for including transposable elements in differential expression analysis of RNA-seq datasets. Bioinformatics 31, 3593–3599. 10.1093/bioinformatics/btv422 26206304 PMC4757950

[B84] Joly-Lopez Z. Bureau T. E. (2018). Exaptation of transposable element coding sequences. Curr. Opin. Genet. Dev. 49, 34–42. 10.1016/j.gde.2018.02.011 29525543

[B85] Jönsson M. E. Ludvik Brattås P. Gustafsson C. Petri R. Yudovich D. Pircs K. (2019). Activation of neuronal genes *via* LINE-1 elements upon global DNA demethylation in human neural progenitors. Nat. Commun. 10, 3182. 10.1038/s41467-019-11150-8 31320637 PMC6639357

[B86] Kamitaki N. Hujoel M. L. A. Mukamel R. E. Gebara E. McCarroll S. A. Loh P.-R. (2024). A sequence of SVA retrotransposon insertions in ASIP shaped human pigmentation. Nat. Genet. 56, 1583–1591. 10.1038/s41588-024-01841-4 39048794 PMC11319198

[B87] Kapitonov V. V. Jurka J. (2005). RAG1 core and V(D)J recombination signal sequences were derived from Transib transposons. PLoS Biol. 3, e181. 10.1371/journal.pbio.0030181 15898832 PMC1131882

[B88] Kapusta A. Kronenberg Z. Lynch V. J. Zhuo X. Ramsay L. Bourque G. (2013). Transposable elements are major contributors to the origin, diversification, and regulation of vertebrate long noncoding RNAs. PLoS Genet. 9, e1003470. 10.1371/journal.pgen.1003470 23637635 PMC3636048

[B89] Karakülah G. Arslan N. Yandım C. Suner A. (2019). TEffectR: an R package for studying the potential effects of transposable elements on gene expression with linear regression model. PeerJ 7, e8192. 10.7717/peerj.8192 31824778 PMC6899341

[B90] Karttunen K. Patel D. Xia J. Fei L. Palin K. Aaltonen L. (2023). Transposable elements as tissue-specific enhancers in cancers of endodermal lineage. Nat. Commun. 14, 5313. 10.1038/s41467-023-41081-4 37658059 PMC10474299

[B91] Kazazian H. H. Moran J. V. (2017). Mobile DNA in health and disease. N. Engl. J. Med. 377, 361–370. 10.1056/NEJMra1510092 28745987 PMC5980640

[B92] Kelley D. Rinn J. (2012). Transposable elements reveal a stem cell-specific class of long noncoding RNAs. Genome Biol. 13, R107. 10.1186/gb-2012-13-11-r107 23181609 PMC3580499

[B93] Kojima K. K. Smit A. F. A. Bao W. Kohany O. Kojima N. F. Jurka T. (2026). Unifying Repbase and Dfam: a new open foundation for transposable element research. Mob. DNA 17, 17. 10.1186/s13100-026-00409-9 PMC13296103 42343465

[B94] Koonin E. V. Krupovic M. (2015). Evolution of adaptive immunity from transposable elements combined with innate immune systems. Nat. Rev. Genet. 16, 184–192. 10.1038/nrg3859 25488578 PMC5898214

[B95] Krainc T. Fuentes A. (2022). Genetic ancestry in precision medicine is reshaping the race debate. Proc. Natl. Acad. Sci. U.S.A. 119, e2203033119. 10.1073/pnas.2203033119 35294278 PMC8944248

[B96] Kunarso G. Chia N.-Y. Jeyakani J. Hwang C. Lu X. Chan Y.-S. (2010). Transposable elements have rewired the core regulatory network of human embryonic stem cells. Nat. Genet. 42, 631–634. 10.1038/ng.600 20526341

[B97] Lander E. S. Linton L. M. Birren B. Nusbaum C. Zody M. C. Baldwin J. (2001). Initial sequencing and analysis of the human genome. Nature 409, 860–921. 10.1038/35057062 11237011

[B98] Levin H. L. Moran J. V. (2011). Dynamic interactions between transposable elements and their hosts. Nat. Rev. Genet. 12, 615–627. 10.1038/nrg3030 21850042 PMC3192332

[B99] Li Y. Lyu R. Chen S. Wang Y. Sun M.-A. (2024). TEENA: an integrated web server for transposable element enrichment analysis in various model and non-model organisms. Nucleic Acids Res. 52, W126–W131. 10.1093/nar/gkae411 38747349 PMC11223789

[B100] Liao W.-W. Asri M. Ebler J. Doerr D. Haukness M. Hickey G. (2023). A draft human pangenome reference. Nature 617, 312–324. 10.1038/s41586-023-05896-x 37165242 PMC10172123

[B101] Linker S. B. Marchetto M. C. Narvaiza I. Denli A. M. Gage F. H. (2017). Examining non-LTR retrotransposons in the context of the evolving primate brain. BMC Biol. 15, 68. 10.1186/s12915-017-0409-z 28800766 PMC5554003

[B102] Liu T. Conesa A. (2025). Profiling the epigenome using long-read sequencing. Nat. Genet. 57, 27–41. 10.1038/s41588-024-02038-5 39779955

[B103] Mallick S. Micco A. Mah M. Ringbauer H. Lazaridis I. Olalde I. (2024). The Allen Ancient DNA Resource (AADR) a curated compendium of ancient human genomes. Sci. Data 11, 182. 10.1038/s41597-024-03031-7 38341426 PMC10858950

[B104] Manolio T. A. Collins F. S. Cox N. J. Goldstein D. B. Hindorff L. A. Hunter D. J. (2009). Finding the missing heritability of complex diseases. Nature 461, 747–753. 10.1038/nature08494 19812666 PMC2831613

[B105] Marnetto D. Mantica F. Molineris I. Grassi E. Pesando I. Provero P. (2018). Evolutionary rewiring of human regulatory networks by waves of genome expansion. Am. J. Hum. Genet. 102, 207–218. 10.1016/j.ajhg.2017.12.014 29357977 PMC5985534

[B106] Martiniano R. Garrison E. Jones E. R. Manica A. Durbin R. (2020). Removing reference bias and improving indel calling in ancient DNA data analysis by mapping to a sequence variation graph. Genome Biol. 21, 250. 10.1186/s13059-020-02160-7 32943086 PMC7499850

[B107] Matsushima W. Planet E. Trono D. (2024). Ancestral genome reconstruction enhances transposable element annotation by identifying degenerate integrants. Cell Genom 4, 100497. 10.1016/j.xgen.2024.100497 38295789 PMC10879028

[B108] McClintock B. (1950). The origin and behavior of mutable loci in maize. Proc. Natl. Acad. Sci. U.S.A. 36, 344–355. 10.1073/pnas.36.6.344 15430309 PMC1063197

[B109] Miao J. Li D. (2026). TEvarSim: a genome simulator for transposable element (TE) variants. PLoS Comput. Biol. 22, e1013933. 10.1371/journal.pcbi.1013933 41616024 PMC12875575

[B110] Miousse I. R. Chalbot M.-C. G. Lumen A. Ferguson A. Kavouras I. G. Koturbash I. (2015). Response of transposable elements to environmental stressors. Mutat. Research/Reviews Mutat. Res. 765, 19–39. 10.1016/j.mrrev.2015.05.003 26281766 PMC4544780

[B111] Misiak B. Ricceri L. Sąsiadek M. M. (2019). Transposable elements and their epigenetic regulation in mental disorders: current evidence in the field. Front. Genet. 10, 580. 10.3389/fgene.2019.00580 31293617 PMC6603224

[B112] Modenini G. Abondio P. Guffanti G. Boattini A. Macciardi F. (2023). Evolutionarily recent retrotransposons contribute to schizophrenia. Transl. Psychiatry 13, 181. 10.1038/s41398-023-02472-9 37244930 PMC10224989

[B113] Modenini G. Abondio P. Sazzini M. Boattini A. (2024). Polymorphic transposable elements provide new insights on high-altitude adaptation in the Tibetan Plateau. Genomics 116, 110854. 10.1016/j.ygeno.2024.110854 38701989

[B114] Modzelewski A. J. Gan Chong J. Wang T. He L. (2022). Mammalian genome innovation through transposon domestication. Nat. Cell Biol. 24, 1332–1340. 10.1038/s41556-022-00970-4 36008480 PMC9729749

[B115] Moran J. V. DeBerardinis R. J. Kazazian H. H. (1999). Exon shuffling by L1 retrotransposition. Science 283, 1530–1534. 10.1126/science.283.5407.1530 10066175

[B116] Mourtzi N. Sertedaki A. Charmandari E. (2021). Glucocorticoid signaling and epigenetic alterations in stress-related disorders. IJMS 22, 5964. 10.3390/ijms22115964 34073101 PMC8198182

[B117] Muotri A. R. Chu V. T. Marchetto M. C. N. Deng W. Moran J. V. Gage F. H. (2005). Somatic mosaicism in neuronal precursor cells mediated by L1 retrotransposition. Nature 435, 903–910. 10.1038/nature03663 15959507

[B118] Nelson M. G. Linheiro R. S. Bergman C. M. (2017). McClintock: an integrated pipeline for detecting transposable element insertions in whole-genome shotgun sequencing data. G3 Genes|Genomes|Genetics 7, 2763–2778. 10.1534/g3.117.043893 28637810 PMC5555480

[B119] Nesse R. M. (2023). Evolutionary psychiatry: foundations, progress and challenges. World Psychiatry 22, 177–202. 10.1002/wps.21072 37159362 PMC10168175

[B120] Nguyen T. M. Alchalabi S. Oluwatoyosi A. Ropri A. S. Herschkowitz J. I. Rosen J. M. (2020). New twists on long noncoding RNAs: from mobile elements to motile cancer cells. RNA Biol. 17, 1535–1549. 10.1080/15476286.2020.1760535 32522127 PMC7567495

[B121] Nurk S. Koren S. Rhie A. Rautiainen M. Bzikadze A. V. Mikheenko A. (2022). The complete sequence of a human genome. Science 376, 44–53. 10.1126/science.abj6987 35357919 PMC9186530

[B122] Ochoa Thomas E. Zuniga G. Sun W. Frost B. (2020). Awakening the dark side: retrotransposon activation in neurodegenerative disorders. Curr. Opin. Neurobiol. 61, 65–72. 10.1016/j.conb.2020.01.012 32092528 PMC7198348

[B123] Okay K. Varış P. Ü. Miral S. Pavlopoulou A. Oktay Y. Karakülah G. (2023). Whole genome analysis of dizygotic twins with autism reveals prevalent Transposon insertion within neuronal regulatory elements: potential implications for disease etiology and clinical assessment. J. Autism Dev. Disord. 53, 1091–1106. 10.1007/s10803-022-05636-6 35759154

[B124] Oliva A. Tobler R. Cooper A. Llamas B. Souilmi Y. (2021). Systematic benchmark of ancient DNA read mapping. Brief. Bioinform 22, bbab076. 10.1093/bib/bbab076 33834210

[B125] Oomen M. E. Torres-Padilla M.-E. (2024). Jump-starting life: balancing transposable element co-option and genome integrity in the developing mammalian embryo. EMBO Rep. 25, 1721–1733. 10.1038/s44319-024-00118-5 38528171 PMC11015026

[B126] Orgel L. E. Crick F. H. (1980). Selfish DNA: the ultimate parasite. Nature 284, 604–607. 10.1038/284604a0 7366731

[B127] Orlando L. Allaby R. Skoglund P. Der Sarkissian C. Stockhammer P. W. Ávila-Arcos M. C. (2021). Ancient DNA analysis. Nat. Rev. Methods Prim. 1, 14. 10.1038/s43586-020-00011-0

[B128] Owen M. J. Williams N. M. (2021). Explaining the missing heritability of psychiatric disorders. World Psychiatry 20, 294–295. 10.1002/wps.20870 34002520 PMC8129850

[B129] O’Neill K. Liao W.-W. Patel A. Hammell M. G. (2018). TEsmall identifies small RNAs associated with targeted inhibitor resistance in Melanoma. Front. Genet. 9, 461. 10.3389/fgene.2018.00461 30349559 PMC6186986

[B130] Patoori S. Barnada S. M. Large C. Murray J. I. Trizzino M. (2022). Young transposable elements rewired gene regulatory networks in human and chimpanzee hippocampal intermediate progenitors. Development 149, dev200413. 10.1242/dev.200413 36052683 PMC9641669

[B131] Percharde M. Lin C.-J. Yin Y. Guan J. Peixoto G. A. Bulut-Karslioglu A. (2018). A LINE1-Nucleolin partnership regulates early development and ESC identity. Cell 174, 391–405.e19. 10.1016/j.cell.2018.05.043 29937225 PMC6046266

[B132] Perna N. T. Batzer M. A. Deininger P. L. Stoneking M. (1992). Alu insertion polymorphism: a new type of marker for human population studies. Hum. Biol. 64, 641–648. 1328024

[B133] Peze-Heidsieck E. Bonnifet T. Znaidi R. Ravel-Godreuil C. Massiani-Beaudoin O. Joshi R. L. (2022). Retrotransposons as a source of DNA damage in neurodegeneration. Front. Aging Neurosci. 13, 786897. 10.3389/fnagi.2021.786897 PMC8764243 35058771

[B134] Piacentini L. Fanti L. Specchia V. Bozzetti M. P. Berloco M. Palumbo G. (2014). Transposons, environmental changes, and heritable induced phenotypic variability. Chromosoma 123, 345–354. 10.1007/s00412-014-0464-y 24752783 PMC4107273

[B135] Pimpinelli S. Piacentini L. (2020). Environmental change and the evolution of genomes: transposable elements as translators of phenotypic plasticity into genotypic variability. Funct. Ecol. 34, 428–441. 10.1111/1365-2435.13497

[B136] Platt R. N. Vandewege M. W. Ray D. A. (2018). Mammalian transposable elements and their impacts on genome evolution. Chromosome Res. 26, 25–43. 10.1007/s10577-017-9570-z 29392473 PMC5857283

[B137] Playfoot C. J. Duc J. Sheppard S. Dind S. Coudray A. Planet E. (2021). Transposable elements and their KZFP controllers are drivers of transcriptional innovation in the developing human brain. Genome Res. 31, 1531–1545. 10.1101/gr.275133.120 34400477 PMC8415367

[B138] Pontis J. Planet E. Offner S. Turelli P. Duc J. Coudray A. (2019). Hominoid-Specific transposable elements and KZFPs Facilitate Human embryonic genome activation and control transcription in naive Human ESCs. Cell Stem Cell 24, 724–735.e5. 10.1016/j.stem.2019.03.012 31006620 PMC6509360

[B139] Prokopov D. Tunbak H. Leddy E. Drylie B. Camera F. Deniz Ö. (2025). Transposable elements as genome regulators in normal and malignant haematopoiesis. Blood Cancer J. 15, 87. 10.1038/s41408-025-01295-9 40328728 PMC12056191

[B140] Prüfer K. (2018). snpAD: an ancient DNA genotype caller. Bioinformatics 34, 4165–4171. 10.1093/bioinformatics/bty507 29931305 PMC6289138

[B141] Rangel S. C. da Silva M. D. Natrielli Filho D. G. Santos S. N. do Amaral J. B. Victor J. R. (2024). HERV-W upregulation expression in bipolar disorder and schizophrenia: unraveling potential links to systemic immune/inflammation status. Retrovirology 21, 7. 10.1186/s12977-024-00640-3 PMC11034070 38644495

[B142] Rangwala S. H. Zhang L. Kazazian H. H. (2009). Many LINE1 elements contribute to the transcriptome of human somatic cells. Genome Biol. 10, R100. 10.1186/gb-2009-10-9-r100 19772661 PMC2768975

[B143] Ravel‐Godreuil C. Znaidi R. Bonnifet T. Joshi R. L. Fuchs J. (2021). Transposable elements as new players in neurodegenerative diseases. FEBS Lett. 595, 2733–2755. 10.1002/1873-3468.14205 34626428

[B144] Ray D. A. Batzer M. A. (2011). Reading TE leaves: new approaches to the identification of transposable element insertions. Genome Res. 21, 813–820. 10.1101/gr.110528.110 21632748 PMC3106314

[B145] Ray D. A. Xing J. Salem A.-H. Batzer M. A. (2006). SINEs of a nearly perfect character. Syst. Biol. 55, 928–935. 10.1080/10635150600865419 17345674

[B146] Reggiardo R. E. Maroli S. V. Kim D. H. (2022). LncRNA biomarkers of inflammation and cancer. Adv. Exp. Med. Biol. 1363, 121–145. 10.1007/978-3-030-92034-0_7 35220568

[B147] Reilly M. T. Faulkner G. J. Dubnau J. Ponomarev I. Gage F. H. (2013). The role of transposable elements in health and diseases of the central nervous system. J. Neurosci. 33, 17577–17586. 10.1523/JNEUROSCI.3369-13.2013 24198348 PMC3818539

[B148] Renaud G. Hanghøj K. Willerslev E. Orlando L. (2017). Gargammel: a sequence simulator for ancient DNA. Bioinformatics 33, 577–579. 10.1093/bioinformatics/btw670 27794556 PMC5408798

[B149] Richter T. A. Aiken A. A. Puracchio M. J. Maganga-Bakita I. Hunter R. G. (2023). Maternal immune activation and enriched environments impact B2 SINE expression in stress sensitive brain regions of rodent offspring. Genes 14, 858. 10.3390/genes14040858 37107616 PMC10137338

[B150] Rishishwar L. Tellez Villa C. E. Jordan I. K. (2015). “Transposable element polymorphisms recapitulate human evolution,” 6. Mob DNA, 21. 10.1186/s13100-015-0052-6 PMC4647816 26579215

[B151] Rishishwar L. Mariño-Ramírez L. Jordan I. K. (2017). Benchmarking computational tools for polymorphic transposable element detection. Brief. Bioinform 18, 908–918. 10.1093/bib/bbw072 27524380 PMC5808724

[B152] Rishishwar L. Wang L. Wang J. Yi S. V. Lachance J. Jordan I. K. (2018). Evidence for positive selection on recent human transposable element insertions. Gene 675, 69–79. 10.1016/j.gene.2018.06.077 29953920

[B153] Rogers R. L. (2015). Chromosomal rearrangements as barriers to genetic homogenization between archaic and modern humans. Mol. Biol. Evol. 32, 3064–3078. 10.1093/molbev/msv204 26399483 PMC5009956

[B154] Roulois D. Loo Yau H. Singhania R. Wang Y. Danesh A. Shen S. Y. (2015). DNA-Demethylating agents target colorectal cancer cells by inducing viral mimicry by endogenous transcripts. Cell 162, 961–973. 10.1016/j.cell.2015.07.056 26317465 PMC4843502

[B155] Rubin J. van Waaij J. Kraft L. Sirén J. Sackett P. W. Renaud G. (2025). SAFARI: pangenome alignment of ancient DNA using purine/pyrimidine encodings. Nar. Genom Bioinform 7, lqaf170. 10.1093/nargab/lqaf170 41383830 PMC12693626

[B156] Saeliw T. Kanlayaprasit S. Thongkorn S. Songsritaya K. Sanannam B. Sae-Lee C. (2023). Epigenetic gene-regulatory loci in Alu elements associated with autism susceptibility in the prefrontal cortex of ASD. IJMS 24, 7518. 10.3390/ijms24087518 37108679 PMC10139202

[B157] Sandroni V. Chaumette B. (2025). Understanding the emergence of schizophrenia in the light of human evolution: new perspectives in genetics. Genes Brain Behav. 24, e70013. 10.1111/gbb.70013 PMC11725983 39801370

[B158] Schloissnig S. Pani S. Ebler J. Hain C. Tsapalou V. Söylev A. (2025). Structural variation in 1,019 diverse humans based on long-read sequencing. Nature 644, 442–452. 10.1038/s41586-025-09290-7 40702182 PMC12350158

[B159] Senft A. D. Macfarlan T. S. (2021). Transposable elements shape the evolution of mammalian development. Nat. Rev. Genet. 22, 691–711. 10.1038/s41576-021-00385-1 34354263

[B160] Shi J. Jia Z. Sun J. Wang X. Zhao X. Zhao C. (2023). Structural variants involved in high-altitude adaptation detected using single-molecule long-read sequencing. Nat. Commun. 14, 8282. 10.1038/s41467-023-44034-z 38092772 PMC10719358

[B161] Sierra P. Durbin R. (2024). Identification of transposable element families from pangenome polymorphisms. Mob. DNA 15, 13. 10.1186/s13100-024-00323-y PMC11202377 38926873

[B162] Sikela J. M. Searles Quick V. B. (2018). Genomic trade-offs: are autism and schizophrenia the steep price of the human brain? Hum. Genet. 137, 1–13. 10.1007/s00439-017-1865-9 29335774 PMC5898792

[B163] Simonson T. S. Yang Y. Huff C. D. Yun H. Qin G. Witherspoon D. J. (2010). Genetic evidence for high-altitude adaptation in Tibet. Science 329, 72–75. 10.1126/science.1189406 20466884

[B164] Smaers J. B. Gómez-Robles A. Parks A. N. Sherwood C. C. (2017). Exceptional evolutionary expansion of prefrontal cortex in great apes and humans. Curr. Biol. 27, 714–720. 10.1016/j.cub.2017.01.020 28162899

[B165] Smith R. J. Jungers W. L. (1997). Body mass in comparative primatology. J. Hum. Evol. 32, 523–559. 10.1006/jhev.1996.0122 9210017

[B166] Spirito G. Filosi M. Domenici E. Mangoni D. Gustincich S. Sanges R. (2023). Exploratory analysis of L1 retrotransposons expression in autism. Mol. Autism 14, 22. 10.1186/s13229-023-00554-5 PMC10303858 37381037

[B167] Storer J. Hubley R. Rosen J. Wheeler T. J. Smit A. F. (2021). The Dfam community resource of transposable element families, sequence models, and genome annotations. Mob. DNA 12, 2. 10.1186/s13100-020-00230-y PMC7805219 33436076

[B168] Su M. Han D. Boyd-Kirkup J. Yu X. Han J.-D. J. (2014). Evolution of alu elements toward enhancers. Cell Rep. 7, 376–385. 10.1016/j.celrep.2014.03.011 24703844

[B169] Tatonetti N. P. Elhadad N. (2021). Fine-scale genetic ancestry as a potential new tool for precision medicine. Nat. Med. 27, 1152–1153. 10.1038/s41591-021-01405-7 34140703

[B170] Techaniyom P. Korsirikoon C. Chitta P. Sae-Lee C. (2025). Regulatory roles of transposable elements on autism molecular neuropathology. Epigenomics 17, 691–699. 10.1080/17501911.2025.2501520 40326043 PMC12233724

[B171] Teresi S. J. Teresi M. B. Edger P. P. (2022). TE Density: a tool to investigate the biology of transposable elements. Mob. DNA 13, 11. 10.1186/s13100-022-00264-4 PMC9004194 35413944

[B172] Turelli P. Playfoot C. Grun D. Raclot C. Pontis J. Coudray A. (2020). Primate-restricted KRAB zinc finger proteins and target retrotransposons control gene expression in human neurons. Sci. Adv. 6, eaba3200. 10.1126/sciadv.aba3200 32923624 PMC7455193

[B173] Vaddadi K. Lin M.-J. Majidian S. Mun T. Langmead B. (2026). Minimizing reference bias with an imputed personalized reference. Genome Res. 36, 740–753. 10.1101/gr.280989.125 PMC13138014 41786604

[B174] Van Bree E. J. Guimarães R. L. F. P. Lundberg M. Blujdea E. R. Rosenkrantz J. L. White F. T. G. (2022). A hidden layer of structural variation in transposable elements reveals potential genetic modifiers in human disease-risk loci. Genome Res. 32, 656–670. 10.1101/gr.275515.121 PMC8997352 35332097

[B175] Van Calker D. Serchov T. (2021). The “missing heritability”—Problem in psychiatry: is the interaction of genetics, epigenetics and transposable elements a potential solution? Neurosci. and Biobehav. Rev. 126, 23–42. 10.1016/j.neubiorev.2021.03.019 33757815

[B176] Venter J. C. Adams M. D. Myers E. W. Li P. W. Mural R. J. Sutton G. G. (2001). The sequence of the human genome. Science 291, 1304–1351. 10.1126/science.1058040 11181995

[B177] Wang Y. Zhao B. Choi J. Lee E. A. (2021). Genomic approaches to trace the history of human brain evolution with an emerging opportunity for transposon profiling of ancient humans. Mob. DNA 12, 22. 10.1186/s13100-021-00250-2 PMC8525043 34663455

[B178] Watkins W. S. Feusier J. E. Thomas J. Goubert C. Mallick S. Jorde L. B. (2020). The simons genome diversity Project: a global analysis of Mobile element diversity. Genome Biol. Evol. 12, 779–794. 10.1093/gbe/evaa086 32359137 PMC7290288

[B179] Witt K. E. Huerta-Sánchez E. (2019). Convergent evolution in human and domesticate adaptation to high-altitude environments. Phil. Trans. R. Soc. B 374, 20180235. 10.1098/rstb.2018.0235 31154977 PMC6560271

[B180] Xia B. Zhang W. Zhao G. Zhang X. Bai J. Brosh R. (2024). On the genetic basis of tail-loss evolution in humans and apes. Nature 626, 1042–1048. 10.1038/s41586-024-07095-8 PMC10901737 38418917

[B181] Xing J. Zhang Y. Han K. Salem A. H. Sen S. K. Huff C. D. (2009). Mobile elements create structural variation: analysis of a complete human genome. Genome Res. 19, 1516–1526. 10.1101/gr.091827.109 19439515 PMC2752133

[B182] Yang H. Sun W. Li J. Zhang X. (2025). Epigenetics factors in schizophrenia: future directions for etiologic and therapeutic study approaches. Ann. Gen. Psychiatry 24, 21. 10.1186/s12991-025-00557-x 40186258 PMC11969811

[B183] Yi X. Liang Y. Huerta-Sanchez E. Jin X. Cuo Z. X. P. Pool J. E. (2010). Sequencing of 50 human exomes reveals adaptation to high altitude. Science 329, 75–78. 10.1126/science.1190371 20595611 PMC3711608

[B184] Zapater L. J. Lewis S. A. Gutierrez R. L. Yamada M. Rodriguez-Fos E. Planas-Felix M. (2026). A transposase-derived gene required for human brain development. Sci. Adv. 12, eadv7530. 10.1126/sciadv.adv7530 PMC12802831 41533792

[B185] Zhang Y. (2024). LncRNA-encoded peptides in cancer. J. Hematol. Oncol. 17, 66. 10.1186/s13045-024-01591-0 PMC11320871 39135098

[B186] Zhou B. Wen S. Wang L. Jin L. Li H. Zhang H. (2017). AntCaller: an accurate variant caller incorporating ancient DNA damage. Mol. Genet. Genomics 292, 1419–1430. 10.1007/s00438-017-1358-5 28836000

[B187] Zhou B. He Y. Chen Y. Su B. (2023). Comparative genomic analysis identifies great–ape–specific structural variants and their evolutionary relevance. Mol. Biol. Evol. 40, msad184. 10.1093/molbev/msad184 PMC10461412 37565562

[B188] Zoonomia Consortium (2020). A comparative genomics multitool for scientific discovery and conservation. Nature 587, 240–245. 10.1038/s41586-020-2876-6 33177664 PMC7759459

